# Changes in Left Ventricular Volumes and Function After Initiation of Sodium–Glucose Cotransporter-2 Inhibitors in Patients with Heart Failure and Predominantly Reduced Ejection Fraction: A Real-World Echocardiographic Study

**DOI:** 10.3390/jcm15218679

**Published:** 2026-11-06

**Authors:** Lucia Tricarico, Michele Granatiero, Roberto Licordari, Elena Costabile, Ginevra Perconti, Savino Cafagna, Florinda Rosa Bonanno, Luca Battisti, Francesca Delia, Massimo Iacoviello, Natale Daniele Brunetti, Giuseppe Dattilo, Michele Correale

**Affiliations:** 1 Cardiology Unit, Emergency and Urgency Department, Policlinico Riuniti University Hospital, 71100 Foggia, Italy; lucia.tricarico.lt@gmail.com (L.T.); michelegranatiero94@gmail.com (M.G.); 2 Department of Biomedical and Dental Sciences and of Morphological and Functional Images, University of Messina, 98125 Messina, Italy; robertolicordari@gmail.com (R.L.); dattimed@gmail.com (G.D.); 3 Department of Medical and Surgical Sciences, University of Foggia, 71122 Foggia, Italy; elenacostabile@gmail.com (E.C.); saviocafagna11@gmail.com (S.C.); lucabattisti1997@gmail.com (L.B.); massimo.iacoviello@gmail.com (M.I.); natale.brunetti@unifg.it (N.D.B.); 4 Department of Clinical and Experimental Medicine, University of Messina, 98125 Messina, Italy; ginevraperconti@gmail.com (G.P.); florinda.bon@gmail.com (F.R.B.); delia.francesca.21@gmail.com (F.D.)

**Keywords:** SGLT2 inhibitor, gliflozin, heart failure, left ventricular remodelling, echocardiography, landmark analysis

## Abstract

**Background**: Sodium–glucose cotransporter-2 inhibitors (SGLT2i) improve outcomes in heart failure (HF), but baseline characteristics associated with left ventricular (LV) changes after their initiation in routine practice are incompletely defined. **Methods**: In this retrospective two-centre study, 101 patients with HF (94 HFrEF, 6 HFmrEF, 1 HFpEF) initiating an SGLT2i underwent echocardiography at baseline and 6 months. For the purposes of this study, LV reverse remodelling was defined as a simultaneous reduction in LV end-diastolic (LVEDV) and end-systolic volume (LVESV); LVESV reductions ≥10% and ≥15% were used as stringent definitions. Associations were explored with Firth logistic regression adjusted for age and baseline LVEF. Death or HF hospitalisation after the 6-month visit was analysed in relation to 6-month changes (landmark analysis). **Results**: Median LVEF increased from 28.8% at baseline to 31.6% at 6 months, and the medians of individual paired changes in LVEDV and LVESV were −13.0% and −16.3% (all *p* < 0.001). The operational definition was met by 74 patients (73.3%) and an LVESV reduction ≥15% by 56 (55.4%). Higher BMI and larger baseline LVEDV were associated with LV volume reduction, and lower TAPSE with LVEF response, LVESV reduction ≥15% and NYHA improvement. No statistically significant association was observed between 6-month changes in LVEF or LV volumes and subsequent death or HF hospitalisation; confidence intervals were wide. **Conclusions**: In this uncontrolled cohort, SGLT2i initiation was followed by modest LV volume reduction and a small LVEF increase. These associations are exploratory and hypothesis-generating.

## 1. Introduction

In heart failure with reduced ejection fraction (HFrEF), disease progression is characterised by adverse remodelling, including increased ventricular volumes and reduced left ventricular ejection fraction (LVEF), which are associated with worse clinical outcomes. These changes are captured by non-invasive parameters such as LVEF and left ventricular end-systolic (LVESV) and end-diastolic volumes (LVEDV) [1].

Reverse cardiac remodelling is a central therapeutic target in HFrEF and a goal of contemporary medical and device therapies [2]. Alongside established treatments with anti-remodelling effects, such as angiotensin-converting enzyme (ACE) inhibitors, angiotensin receptor blockers (ARBs), β-blockers, mineralocorticoid receptor antagonists (MRAs) and angiotensin receptor–neprilysin inhibitors (ARNI), sodium–glucose cotransporter-2 inhibitors (SGLT2i) improve cardiovascular outcomes.

SGLT2i reduce the risk of worsening heart failure and heart failure hospitalisation, with consistent benefits irrespective of diabetes status, in patients with reduced, mildly reduced and preserved LVEF [3,4,5,6]. Although the mechanisms underlying these benefits are not fully elucidated [7], experimental and clinical data suggest that SGLT2i may attenuate adverse cardiac remodelling through favourable haemodynamic changes, reduction in myocardial fibrosis and inflammation, modulation of sympathetic activity and improvements in cardiac metabolism [8,9,10,11,12,13,14,15].

Several studies have suggested an association between SGLT2i and reverse cardiac remodelling [16,17], but evidence remains limited and partly conflicting [18,19]. Determinants of reverse remodelling in contemporary HFrEF have been investigated in large cohorts; for example, in a real-world analysis of 3628 patients, Furquim et al. identified clinical and echocardiographic characteristics associated with sustained recovery of LV function [20]. More recently, advanced echocardiographic phenotyping has been applied to patients receiving SGLT2i, including RV free-wall strain in the Empire HF trial [21], RV–pulmonary arterial coupling in the RIVED-HF registry [22] and multi-chamber remodelling in a real-world cohort [23]. Fewer data are available on the baseline clinical and echocardiographic characteristics associated with LV structural and functional changes specifically after SGLT2i initiation in routine practice, particularly when LV, right ventricular (RV) and strain-derived parameters are considered together.

The present study therefore aimed to describe 6-month changes in clinical and echocardiographic parameters after SGLT2i initiation in a real-world population with predominantly reduced LVEF, and to explore which commonly assessed baseline characteristics are associated with LV volume reduction, LVEF increase and New York Heart Association (NYHA) class improvement. As a secondary, exploratory aim, we assessed the association between 6-month changes and subsequent death or heart failure hospitalisation.

## 2. Materials and Methods

### 2.1. Study Design and Population

**Figure 1:**
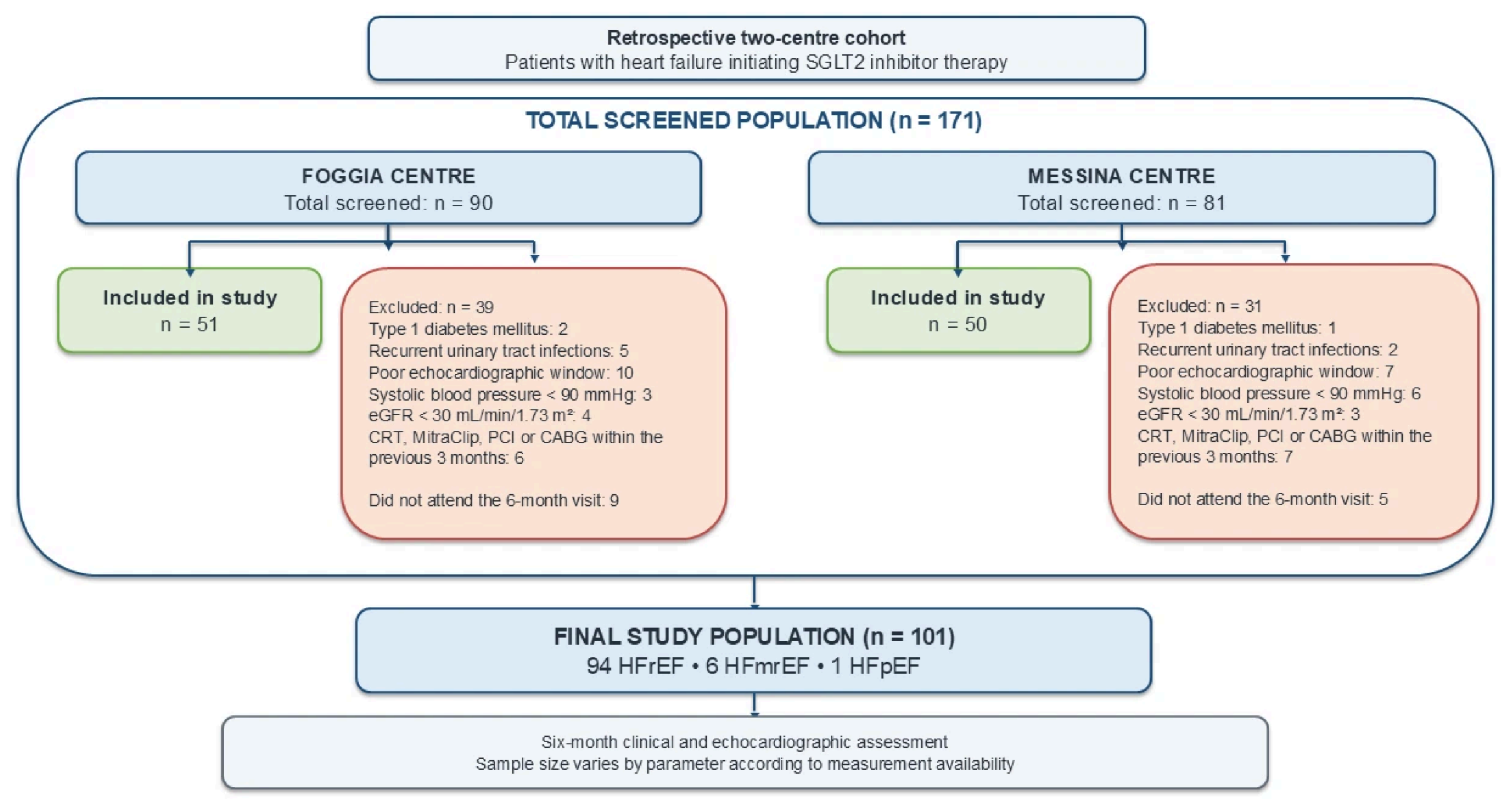
Patient selection and study flow at the two participating centres. CRT: cardiac resynchronisation therapy; CABG: coronary artery bypass grafting; eGFR: estimated glomerular filtration rate; PCI: percutaneous coronary intervention.

This was a retrospective observational study conducted at two centres: the Section of Cardiology, Department of Biomedical and Dental Sciences and Morphofunctional Imaging, University of Messina, Messina, Italy, and the Cardiology Unit, Emergency and Urgency Department, Policlinico Riuniti University Hospital, Foggia, Italy. Consecutive outpatients with a confirmed diagnosis of heart failure, followed at the dedicated heart failure clinics of the two centres, who initiated an SGLT2i as add-on to optimised medical treatment from September 2020 to February 2023 were enrolled. Inclusion required survival and attendance at the 6-month clinical and echocardiographic visit. The selection of patients at each centre is shown in Figure 1. Because only one patient had heart failure with preserved ejection fraction (HFpEF; LVEF ≥ 50%) and six had heart failure with mildly reduced ejection fraction (HFmrEF; LVEF 40–49%), these subgroups were not analysed separately; all analyses were repeated after restriction to patients with HFrEF.

Background therapy was considered optimised when patients were receiving guideline-directed drugs at the maximally tolerated doses according to the treating physician before SGLT2i initiation. Background therapy at baseline and at 6 months is reported in Table 1 and Section 3.7.

### 2.2. Exclusion Criteria

Exclusion criteria included type 1 diabetes mellitus, recurrent urinary tract infections, poor echocardiographic window, systolic blood pressure < 90 mmHg, estimated glomerular filtration rate (eGFR) < 30 mL/min/1.73 m^2^, and implantation of a cardiac resynchronisation therapy (CRT) device, MitraClip, percutaneous coronary intervention, or coronary artery bypass grafting within the previous 3 months; patients with a CRT device implanted more than 3 months before enrolment were therefore eligible.

### 2.3. Clinical Assessment and Echocardiography

Medical history, heart rate, systolic blood pressure, body mass index (BMI), NYHA functional class and medications were recorded. All patients underwent assessment of left and right ventricular function by conventional echocardiography, tissue Doppler imaging and strain echocardiography under resting conditions at baseline and after 6 months of SGLT2i therapy. In the parasternal long-axis view, interventricular septal thickness (IVS), LV posterior wall thickness (LVPW) and LV end-diastolic diameter (LVEDD) were measured, and LV mass was calculated and indexed to body surface area (BSA). In apical four- and two-chamber views, LVEDV and LVESV were measured and LVEF was calculated using the biplane method of discs; volumes and maximal left atrial volume (LAV) were indexed to BSA.

Peak E- and, when present, A-wave velocities of transmitral flow were measured with pulsed-wave Doppler and used to calculate the E/A ratio. Peak e′ velocity at the septal and lateral mitral annulus were measured with pulsed-wave tissue Doppler imaging and used to calculate the E/e′ ratio. RV basal diameter, tricuspid annular plane systolic excursion (TAPSE) and peak tricuspid regurgitant velocity were measured; the latter, together with inferior vena cava diameter and collapsibility, was used to estimate systolic pulmonary arterial pressure (sPAP). Speckle-tracking echocardiography was used to measure LV global longitudinal strain (GLS), left atrial reservoir strain (LARS), RV free-wall strain (RVFWS) and RV global longitudinal strain (RVGLS). Transthoracic echocardiography was performed using an EPIC 7C ultrasound system with an X5-1 transducer ( Philips EPIC 7C system Reedsville, Pennsylvania (USA)), and speckle-tracking analysis was conducted using QLab 10 software. LARS and RV strain analyses were available in 51 patients, because the speckle-tracking analysis software was not available for the analysis of all patients.

Baseline and follow-up examinations were analysed by the same operators, who were blinded to the time point of the examination and to clinical outcomes. All measurements were averaged over five cardiac cycles, both in sinus rhythm and in atrial fibrillation.

### 2.4. Definitions

Because no universally accepted definition of reverse remodelling exists [24], for the purposes of this study, LV reverse remodelling was defined a priori as a simultaneous reduction in LVEDV and LVESV between baseline and 6 months (operational definition). Because this definition does not require a minimum change and may be influenced by test–retest variability of echocardiographic volumes [25], two more stringent definitions were applied in sensitivity analyses: a relative LVESV reduction ≥10% and a relative LVESV reduction ≥ 15% [24]. Absolute and percentage changes in LVEDV and LVESV are also reported.

LVEF response was categorised as response (ΔLVEF ≥5 percentage points), partial response (ΔLVEF 0–4.9 points) or no response (ΔLVEF <0). Functional improvement was defined as a reduction of ≥1 NYHA class. Patients fulfilling both ΔLVEF ≥5 points and NYHA improvement were described as combined echocardiographic and functional responders; this study-specific category was considered exploratory. LVEF categories followed current guideline definitions: HFrEF (<40%), HFmrEF (40–49%) and HFpEF (≥50%) [2]. RV–pulmonary arterial coupling was estimated non-invasively as the TAPSE/sPAP ratio, and eGFR was calculated with the 2021 race-free CKD-EPI creatinine equation.

The clinical endpoint of this exploratory analysis was the composite of all-cause death or heart failure hospitalisation after the 6-month visit; its two components were also analysed separately. Non-fatal myocardial infarction or percutaneous coronary intervention, incident atrial fibrillation (AF) and ventricular arrhythmias (non-sustained ventricular tachycardia, ventricular tachycardia, or ventricular fibrillation) were also recorded and are reported descriptively. Incident AF was defined as a first documented episode of AF in patients without a history of AF; AF recurrences in patients with a known history of AF were not counted as AF events. Ventricular arrhythmias were assessed only in patients with available device telemetry or Holter monitoring. A broader composite of all these events, defined to capture both heart failure-related and arrhythmic events, was analysed as a supplementary endpoint, together with the same composite excluding ventricular arrhythmias. For each endpoint, each patient contributed only the first event.

### 2.5. Statistical Analysis

Continuous variables are reported as median (interquartile range, IQR) and categorical variables as counts and percentages. Paired comparisons between baseline and 6 months were performed with the Wilcoxon signed-rank test for continuous and ordinal variables and McNemar’s test for dichotomous variables, using complete cases for each variable pair; the number of patients contributing to each comparison is reported, and the extent of missing data is shown in Table S1. Drug doses were compared in patients receiving the same drug at both time points; β-blocker doses were expressed as a percentage of the guideline target dose of the respective molecule.

In response to the risk of overfitting associated with univariable *p*-value screening, the modelling strategy was revised. Associations between baseline characteristics and each binary response endpoint were estimated with Firth penalised-likelihood logistic regression, which reduces small-sample bias and provides finite estimates in the presence of sparse data or separation [26]. Each candidate characteristic was examined unadjusted and in a parsimonious model adjusted for age and baseline LVEF (three parameters), without data-driven variable selection. LV GLS was analysed as its absolute value (|GLS|), so that odds ratios (ORs) >1 indicate an association with better baseline longitudinal function. Continuous variables were scaled to clinically interpretable increments. All regression models were fitted on complete cases for the variables included, without imputation; for each model we report the number of patients and events analysed, and Table S2 compares the events per parameter of the original and revised strategies. Multicollinearity was assessed with variance inflation factors (VIFs) and a Spearman correlation matrix (Figure S1), and departure from linearity was tested by comparing linear terms with natural cubic splines (3 degrees of freedom) using likelihood-ratio tests (Table S3). The stability of key associations was assessed with 1000 bootstrap resamples (Table S4). Internal validation of the main logistic and Cox models was performed with 500 bootstrap resamples, and apparent and optimism-corrected C-statistics and calibration slopes are reported (Table S5).

Because dichotomised change outcomes are susceptible to regression to the mean and mathematical coupling with the baseline value, we additionally used analysis of covariance (ANCOVA), modelling the 6-month value as a function of the baseline value of the same parameter, the characteristic of interest and age [27]; LV volumes were log-transformed so that estimates are expressed as percentage differences in the 6-month volume. Sensitivity analyses were performed after restriction to patients with HFrEF (n = 94) and after exclusion of patients with CRT-D (n = 82).

Clinical outcomes were analysed using the 6-month visit as time origin, i.e., as a 6-month landmark analysis, so that 6-month response status was known for all patients at the start of follow-up [28]; patients with non-fatal clinical events between SGLT2i initiation and the 6-month visit remained eligible, such events were not systematically collected and were not counted, and the endpoint was the first event occurring after the 6-month visit, irrespective of any event before this visit. Patients without events were censored at the date of last contact; completeness of follow-up was assessed with the reverse Kaplan–Meier method. Associations between 6-month changes and subsequent events were assessed with Cox proportional hazards regression adjusted for age and baseline LVEF. Changes were analysed as continuous variables (change in LVEF per 5 percentage points; relative changes in LVESV and LVEDV, computed as the log-transformed ratio of the 6-month to the baseline value and expressed per 10% reduction) and according to the response definitions. Associations between baseline characteristics and outcomes were assessed univariably and in a multivariable model including age, baseline LVEF and NYHA class. The proportional hazards assumption was verified with Schoenfeld residuals. For the supplementary composite endpoint, event-free survival was also estimated with the Kaplan–Meier method and compared with the log-rank test. Results are expressed as ORs or hazard ratios (HRs) with 95% confidence intervals (CIs). All tests were two-sided with *p* < 0.05 considered statistically significant; given the number of comparisons, all associations are regarded as exploratory. The operational definition of reverse remodelling, the LVEF and NYHA response categories and the broader composite endpoint were those of the original analysis; the choice of death or heart failure hospitalisation as the main clinical endpoint, the analyses of continuous changes, the LVESV thresholds, the Firth models adjusted for age and baseline LVEF, the ANCOVA models, the landmark Cox models, and the sensitivity, component and internal validation analyses were specified for the revised analysis, after the initial examination of the data, and should be considered post hoc. Analyses were performed in R (version ≥4.3; R Foundation for Statistical Computing, Vienna, Austria) using the survival, brglm2 and car packages.

## 3. Results

### 3.1. Study Population

Of 171 patients screened at the two centres, 56 met at least one exclusion criterion and 14 were lost to follow-up because they did not respond to the recall for the 6-month visit (Figure 1). The study therefore included 101 patients: 94 (93.1%) with HFrEF, 6 (5.9%) with HFmrEF and 1 (1.0%) with HFpEF. Dapagliflozin 10 mg was initiated in 96 patients (95.0%) and empagliflozin 10 mg in 5 (5.0%). Median age was 64 years (IQR 51–71) and 79 patients (78%) were men. At SGLT2i initiation, all patients were receiving a β-blocker, 67 (66%) ARNI, 22 (22%) an ACE inhibitor, 12 (12%) an ARB and 80 (79%) an MRA; 79 patients (78%) were receiving a β-blocker, an MRA and a renin–angiotensin system inhibitor or ARNI, and thus quadruple therapy after SGLT2i initiation. Nineteen patients (19%) had a CRT-D implanted before enrolment. Baseline characteristics are reported in Table 1. Median clinical follow-up after the 6-month visit was 32 months (IQR 25–33).

### 3.2. Clinical and Echocardiographic Changes at 6 Months

Paired comparisons are reported in Table 2 together with the number of patients contributing to each comparison. Median LVEF increased from 28.8% (IQR 24.4–36.0) at baseline to 31.6% (25.6–38.0) at 6 months (*p* < 0.001), and LVEDV, LVEDV index, LVESV and LVESV index decreased (all *p* < 0.001). The median of individual paired changes was −28.0 mL (IQR −48.6 to −2.2) for LVEDV, corresponding to −13.0% (−20.9 to −0.9), and −24.0 mL (−42.0 to −5.5) for LVESV, corresponding to −16.3% (−24.7 to −4.8); the median paired LVEF change was +2.3 percentage points (1.0 to 4.8) (Section 3.3). Median LV GLS, available in 99 patients, improved from −8.9% (−11.9 to −6.7) to −9.9% (−12.4 to −7.2) (*p* = 0.003). LAV decreased (*p* < 0.001; n = 96), whereas the change in LAV index was not significant (*p* = 0.104). Diastolic dysfunction grade (*p* = 0.029) and E/A ratio (*p* < 0.001) decreased.

Median sPAP decreased from 33 mmHg (24–40) to 31 mmHg (23–35) (*p* < 0.001; n = 69), whereas TAPSE did not change significantly (20.3 vs. 21.0 mm; *p* = 0.135; n = 95); accordingly, the increase in the TAPSE/sPAP ratio (*p* < 0.001; n = 68) was driven predominantly by the reduction in estimated sPAP. Small increases in IVS thickness (*p* = 0.001) and LV mass (*p* = 0.001) were observed, without significant change in LV mass index (*p* = 0.560). Haemoglobin decreased (*p* = 0.026) and creatinine increased slightly (*p* = 0.048), without a significant change in eGFR (*p* = 0.062). In the subgroup with LA and RV strain analysis (n = 51), changes in LARS, RVFWS and RVGLS were not significant (Table 3). Results were consistent after restriction to patients with HFrEF and after exclusion of patients with CRT-D (Table S6), although the change in GLS did not reach statistical significance in the latter subgroup (*p* = 0.057).

### 3.3. LV Volume Reduction, LVEF Response and NYHA Class Change

**Figure 2:**
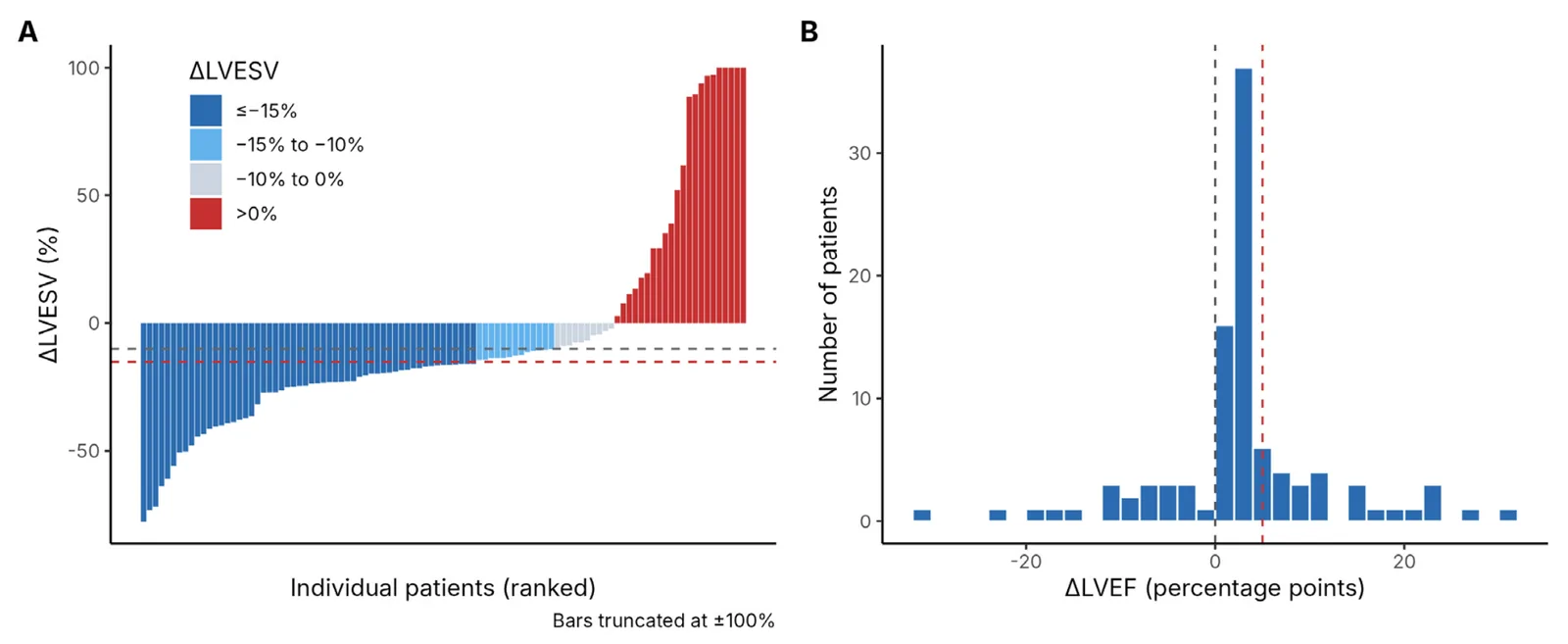
Individual changes from baseline to 6 months. (**A**) Waterfall plot of the percentage change in LVESV; dashed lines indicate −10% and −15% (bars truncated at ±100%). (**B**) Distribution of ΔLVEF; dashed lines indicate ΔLVEF = 0 and ΔLVEF = 5 percentage points.

**Figure 3:**
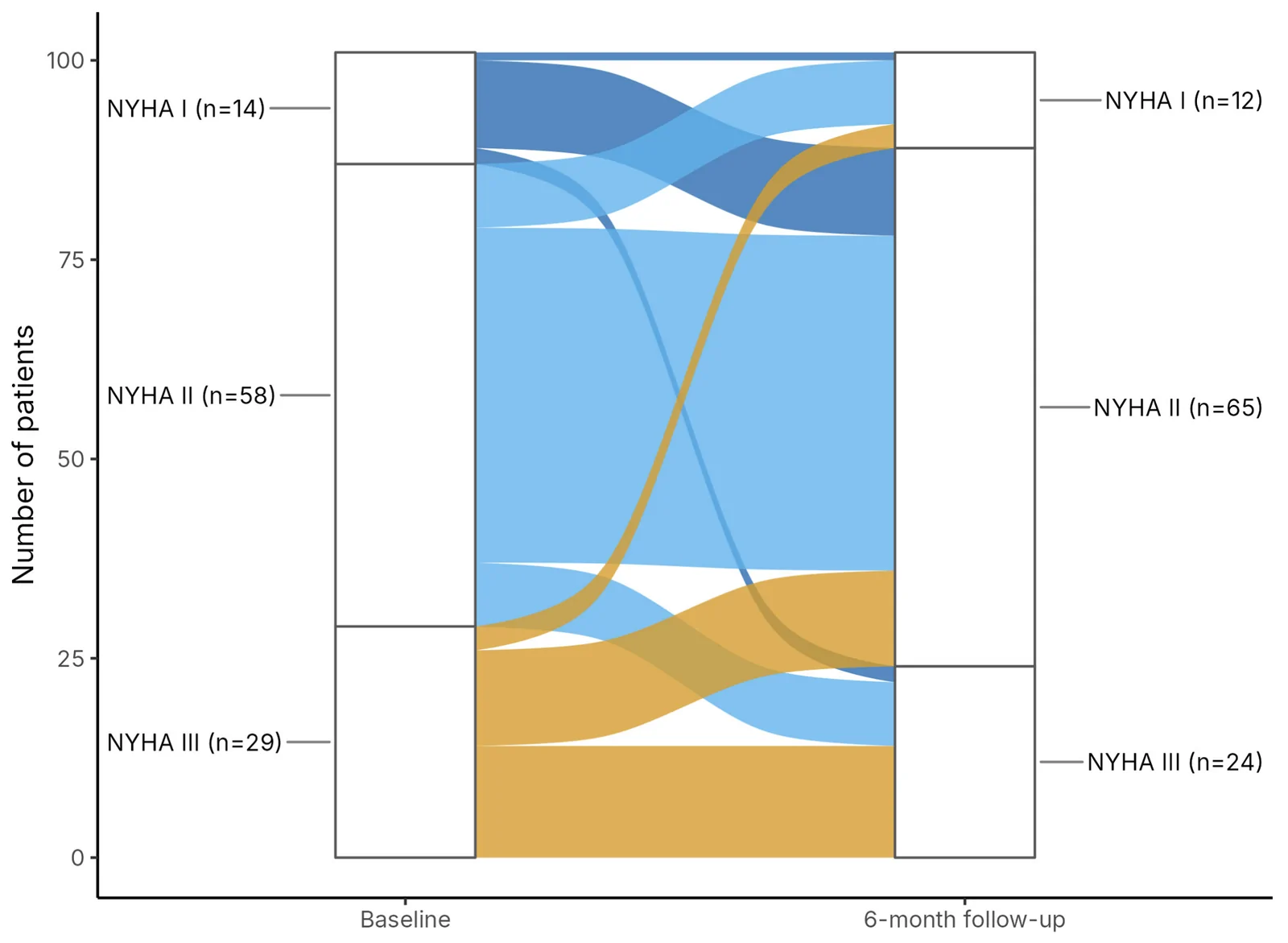
NYHA functional class at baseline and at 6 months.

The operational definition of LV reverse remodelling was fulfilled by 74 patients (73.3%). An LVESV reduction ≥10% occurred in 69 patients (68.3%) and ≥15% in 56 (55.4%); of the 74 patients meeting the operational definition, 68 (91.9%) also had an LVESV reduction ≥10% and only one had a reduction <5% (Table 4 and Figure 2A). Twenty-four patients (23.8%) were LVEF responders, 57 (56.4%) partial responders and 20 (19.8%) non-responders (Figure 2B). Twenty-three patients (22.8%) improved by ≥1 NYHA class, 57 (56.4%) remained stable and 21 (20.8%) worsened (Figure 3). Eight patients (7.9%) were combined echocardiographic and functional responders.

**Figure 4:**
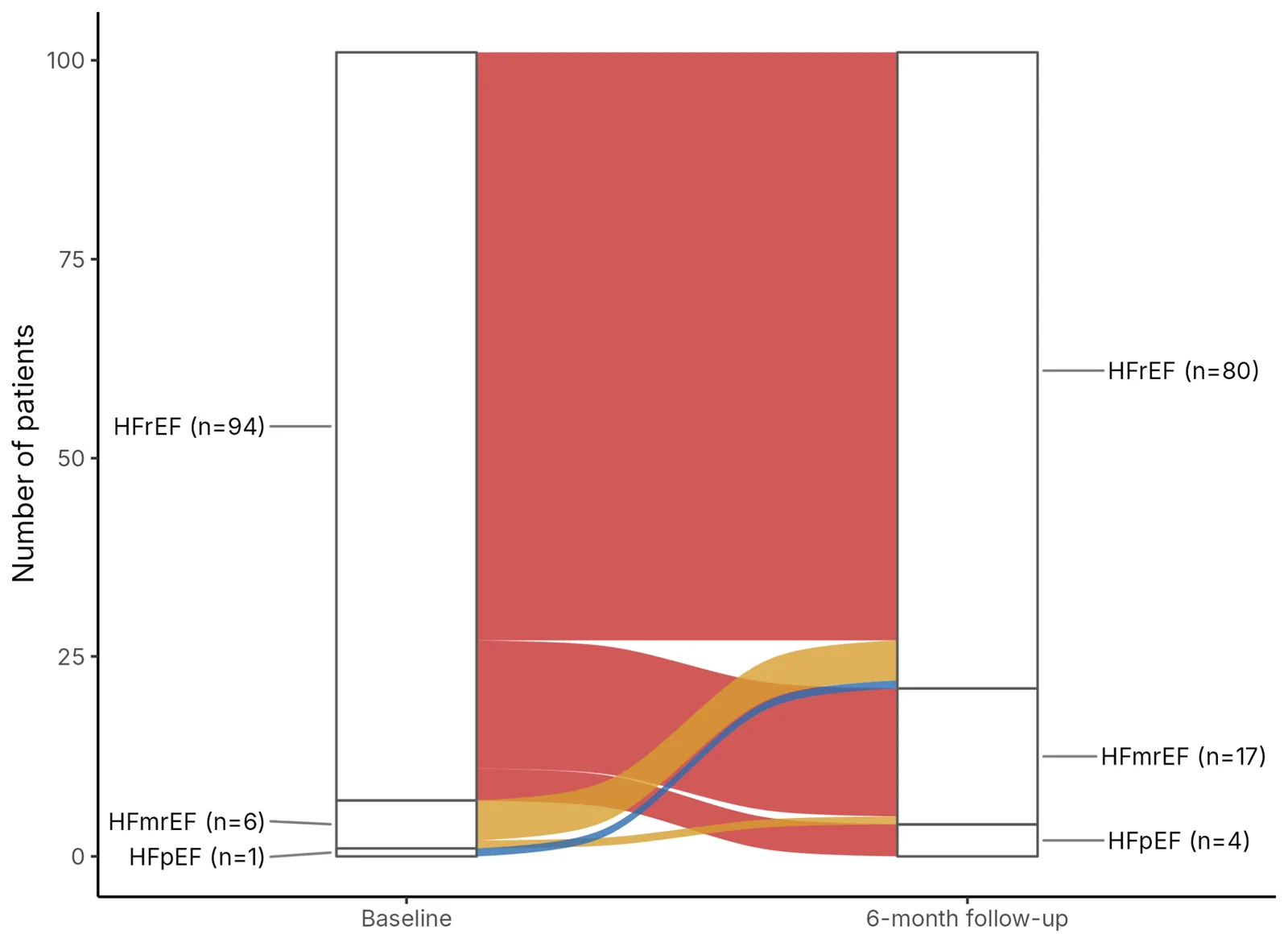
LVEF category at baseline and at 6 months. HFrEF: LVEF < 40%; HFmrEF: 40–49%; HFpEF: ≥50%.

At 6 months, 20 patients (19.8%) moved to a higher LVEF category and 6 (5.9%) to a lower category (net upward reclassification 13.9%); 74 of the 94 patients with HFrEF at baseline (78.7%) remained in the HFrEF category (Figure 4).

### 3.4. Baseline Characteristics Associated with LV Volume Reduction

With the revised strategy, the events per estimated parameter increased from 2.1–3.0 in the original multivariable models to 7.7–9.0, and the maximum VIF decreased from 10.9 to ≤2.9 (Table S2). After adjustment for age and baseline LVEF, the operational definition of LV reverse remodelling was associated with higher BMI (OR 1.30 per kg/m^2^, 95% CI 1.09–1.56), larger baseline LVEDV (OR 1.37 per 10 mL, 1.18–1.60) and larger LAV (OR 1.23 per 10 mL, 1.06–1.43); lower baseline LVEF was associated with this endpoint in the age-adjusted model (OR 0.53 per 5%, 0.37–0.76) (Table 5). Anaemia was not significantly associated with the endpoint (adjusted OR 2.19, 0.66–7.22), and the association with GLS observed in unadjusted analysis (OR 0.82 per 1% higher |GLS|, 0.71–0.95) was not maintained after adjustment (OR 0.94, 0.76–1.16). In bootstrap analysis, the associations of BMI and LVEDV were in the same direction in 100% of resamples and statistically significant in 92% and 100%, respectively (Table S4). In internal validation of the main logistic models, optimism was small, with optimism-corrected C-statistics ranging from 0.60 to 0.89 and calibration slopes from 0.81 to 1.01 (Table S5).

Using the stringent definition (LVESV reduction ≥15%), higher BMI (OR 1.34, 1.15–1.55), larger baseline LVEDV (OR 1.09, 1.01–1.18) and lower TAPSE (OR 0.81 per mm, 0.71–0.92) were associated with the endpoint, whereas the association with baseline LVEF was not significant (Table S7); results with the ≥10% threshold are shown in Table S8. In ANCOVA models of the 6-month LV volumes adjusted for the baseline value, better baseline GLS was associated with smaller 6-month LVESV (−5.6% per 1% higher |GLS|, 95% CI −8.8 to −2.3), whereas higher TAPSE, higher sPAP and higher NYHA class were associated with larger 6-month LVESV; BMI was not independently associated with the 6-month volumes (Table S9). Associations were consistent in patients with HFrEF and after exclusion of patients with CRT-D (Table S6).

### 3.5. Baseline Characteristics Associated with LVEF Response and NYHA Improvement

After adjustment for age and baseline LVEF, LVEF response was associated with lower baseline TAPSE (OR 0.66 per mm, 0.53–0.81), higher sPAP (OR 1.31 per 5 mmHg, 1.01–1.70), lower systolic blood pressure (OR 0.67 per 10 mmHg, 0.47–0.95), smaller baseline LVEDV (OR 0.82 per 10 mL, 0.74–0.91) and LAV (OR 0.85 per 10 mL, 0.74–0.96) and better baseline GLS (OR 1.47 per 1% higher |GLS|, 1.15–1.87) (Table 6). In ANCOVA, better baseline GLS was associated with higher 6-month LVEF (+1.3 points per 1% higher |GLS|, 0.6–1.9) and higher TAPSE with lower 6-month LVEF (−1.2 points per mm, −1.5 to −0.9) (Table S9). Diabetes was not significantly associated with LVEF response (OR 0.22, 0.04–1.33).

NYHA improvement was associated with higher baseline NYHA class (OR 5.97, 2.12–16.78), lower baseline LVEF (OR 0.65 per 5%, 0.44–0.98), lower TAPSE (OR 0.82 per mm, 0.71–0.95) and higher sPAP (OR 1.38 per 5 mmHg, 1.04–1.83) (Table 7). Among combined responders (n = 8), only unadjusted analyses were performed; higher NYHA class, higher E/e′, lower TAPSE and greater mitral regurgitation were associated with this category (Table S10).

### 3.6. Clinical Outcomes After the 6-Month Visit

During a median follow-up of 32 months (IQR 25–33) after the 6-month visit (reverse Kaplan–Meier median 33 months, 95% CI 32–35), death or heart failure hospitalisation occurred in 30 patients (29.7%): all-cause death in 14 (13.9%) and heart failure hospitalisation in 18 (17.8%). Non-fatal myocardial infarction/percutaneous coronary intervention occurred in 2 patients (2.0%), incident AF in 14 of 68 patients without a history of AF (20.6%) and ventricular arrhythmias in 14 of 55 monitored patients (25.5%) (Table 8A); AF recurred in 3 of 33 patients with a history of AF.

After adjustment for age and baseline LVEF, no statistically significant association was observed between the change in LVEF (HR 0.87 per 5-point increase, 0.68–1.12), the relative change in LVESV (HR 1.00 per 10% reduction, 0.92–1.09) or the relative change in LVEDV (HR 1.01 per 10% reduction, 0.92–1.12) and death or heart failure hospitalisation, and none of the response definitions was significantly associated with this endpoint (Table 8B). The confidence intervals were wide and compatible with clinically relevant associations in either direction. In analyses of the individual components, a larger LVEF increase was associated with lower all-cause mortality (HR 0.64 per 5-point increase, 0.43–0.94; 14 deaths), and no patient with NYHA improvement died, whereas NYHA improvement was associated with a higher rate of heart failure hospitalisation (HR 4.20, 1.53–11.52). Given the small number of events, these component analyses are hypothesis-generating.

Among baseline characteristics, ischaemic aetiology was associated with death or heart failure hospitalisation in univariable analysis (HR 2.39, 1.16–4.92), whereas age, baseline LVEF (HR 1.15 per 5%, 0.86–1.53) and NYHA class were not significantly associated with this endpoint in the multivariable model (Table S11), which showed limited discrimination (optimism-corrected C-statistic 0.46; Table S5).

The broader composite endpoint occurred in 47 patients (46.5%) (Table S12). Higher baseline LVEF was associated with this endpoint (HR 1.31 per 5%, 1.05–1.64) (Table S13), mainly through incident AF (HR 1.57 per 5%, 1.03–2.39) (Table S14), and event-free survival did not differ according to LVEF response, NYHA response or the operational definition of LV reverse remodelling (Figures S2–S4; Tables S15 and S16).

### 3.7. Background Therapy

At the group level, the proportions of patients receiving each drug class did not change significantly between baseline and 6 months (Table 9); however, individual treatment changes were frequent: 45 patients (44.6%) had at least one documented initiation or discontinuation of an MRA, ACE inhibitor, ARB or ARNI (no changes in β-blocker use), and ARNI status at 6 months was unknown in 33 patients. Doses of loop diuretics, MRAs and β-blockers (expressed as percentage of target dose: median 25% (IQR 12–50) vs. 38% (12–75), *p* = 0.097; n = 76 receiving the same molecule at both time points) did not differ significantly. In the 26 patients with complete therapy data and no documented change in disease-modifying therapy, LVEDV and LVESV decreased (median paired changes −12.6% and −16.1%; *p* = 0.029 and *p* = 0.022), whereas the LVEF change was not significant (+ 2.0 points; *p* = 0.253) (Table S17).

## 4. Discussion

In this two-centre real-world cohort of patients with predominantly reduced LVEF, SGLT2i initiation on top of background therapy was followed at 6 months by a modest reduction in LV volumes, a small increase in LVEF and an improvement in LV GLS. LV volume reduction was frequent according to our operational definition and remained common with stricter thresholds based on relative LVESV reduction. Larger baseline LV size and higher BMI were associated with LV volume reduction across the binary definitions, whereas lower TAPSE was associated with LVEF response, LVESV reduction ≥15% and NYHA improvement. No statistically significant association was observed between six-month response and subsequent clinical events, although the confidence intervals were wide. Because the study had no control group, these findings describe changes following SGLT2i initiation rather than effects attributable to SGLT2 inhibition.

The direction and magnitude of the observed changes are consistent with randomised data and meta-analyses. In the Empire HF cardiac magnetic resonance substudy, empagliflozin reduced LVESV, LVEDV and left atrial volume indexes after 12 weeks without a significant change in LVEF [29], and SUGAR-DM-HF showed reductions in LVESV and LVEDV indexes with empagliflozin compared with placebo [30]. Meta-analyses have reported reductions in LV volumes with a modest increase in LVEF, whereas evidence regarding GLS is less consistent [31,32], and multi-chamber reverse remodelling after SGLT2i initiation has recently been described in a real-world cohort [23]. Our previous studies in patients receiving contemporary neurohormonal therapy similarly reported improvements in LV dimensions, volumes, LVEF and biventricular function after ARNI and SGLT2i treatment or after switching to SGLT2i [33,34].

The interpretation of within-patient change in an uncontrolled cohort requires caution. Although the overall proportions of patients receiving each drug class did not change significantly, stable group-level prescribing does not establish stability of treatment within individual patients: 45 patients had at least one documented initiation or discontinuation of a disease-modifying drug, and ARNI status at 6 months was incomplete. These individual treatment changes, as well as changes in adherence or dose titration, could have contributed to the echocardiographic findings; LV volumes also decreased in the small subgroup without documented treatment changes, but this analysis had limited power. Recent initiation of other disease-modifying drugs before enrolment may also have played a role. Contemporary HFrEF management relies on multidrug therapy, and an observational design cannot separate the contribution of each drug class. This also applies to β-blockers, whose prognostic role is being reconsidered in specific ischaemic phenotypes and in the modern therapeutic setting [35]. The changes observed in our cohort should therefore be interpreted as multidrug reverse remodelling occurring after SGLT2i initiation, rather than as an isolated effect of SGLT2i.

The response definitions also have methodological limitations. Our operational definition of LV reverse remodelling did not require a minimum change, and the test–retest variability of two-dimensional echocardiographic volumes is substantial [25]. However, more than 90% of patients fulfilling this definition also had an LVESV reduction ≥10%, and 55% of the cohort met the ≥15% threshold commonly used in the literature [24]. Baseline values predicting their own subsequent change are prone to regression to the mean and mathematical coupling; larger ventricles offer greater numerical opportunity for volume reduction. For this reason, we complemented the dichotomous endpoints with ANCOVA models of the 6-month values [27]. The association of larger LVEDV with LV volume reduction may be partly explained by these effects.

The associations of lower baseline TAPSE and higher sPAP with LVEF response and NYHA improvement are consistent with the hypothesis that patients with a greater haemodynamic burden have more room for improvement. Nevertheless, these associations may also reflect regression to the mean, and the improvement in the TAPSE/sPAP ratio was driven predominantly by the reduction in estimated sPAP, with no significant change in TAPSE. We therefore describe this finding as an improvement in the non-invasive TAPSE/sPAP estimate of RV–pulmonary arterial coupling, driven mainly by lower estimated pulmonary pressure, rather than as evidence of recovery of RV systolic function. In the RIVED-HF registry, dapagliflozin was associated with lower pulmonary artery systolic pressure and better TAPSE/sPAP coupling than standard therapy [22], whereas in the Empire HF trial empagliflozin had no overall effect on RV free-wall strain [21], in keeping with the absence of significant RV strain changes in our subgroup. In ANCOVA, higher baseline TAPSE was associated with larger 6-month LV volumes and lower 6-month LVEF, which is directionally consistent with the logistic models.

After sparse-data bias and overfitting were addressed with penalised regression and parsimonious adjustment, anaemia was not significantly associated with LV volume reduction (adjusted OR 2.19, 0.66–7.22). Better baseline GLS was associated with LVEF response and with smaller 6-month LVESV. Preserved longitudinal function may therefore indicate residual contractile reserve. The association of higher BMI with LV volume reduction in logistic models was not confirmed in ANCOVA and may reflect confounding by body size or indexation; it should be considered hypothesis-generating.

With regard to clinical outcomes, death or heart failure hospitalisation occurred in 29.7% of patients during follow-up after the 6-month visit, and no statistically significant association was observed between 6-month changes in LVEF or LV volumes, analysed either as continuous variables or according to the response definitions, and this endpoint. Given the limited sample size and the wide confidence intervals, these results do not establish that 6-month improvement lacks prognostic relevance. The inverse association between the LVEF increase and all-cause death is in keeping with the prognostic relevance of LVEF improvement in HFrEF [24], but it was based on 14 deaths. The association between NYHA improvement and heart failure hospitalisation is counterintuitive and may reflect the worse baseline functional class of patients who improved (15 of 23 were in NYHA class III) and regression to the mean. The broader composite endpoint, which included arrhythmic events, was associated with higher baseline LVEF, mainly through incident AF; because causes of death were not available, the relative contribution of arrhythmic and pump-failure mortality could not be assessed. Our study was designed to investigate structural and functional change rather than clinical outcomes, and larger prospective studies are needed to determine whether changes in LV volumes after SGLT2i initiation translate into improved long-term prognosis.

### Limitations

Several limitations should be acknowledged. First, the retrospective observational design and the absence of a contemporaneous untreated control group prevent causal inference; background therapy, natural recovery, changes in loading conditions, survivor selection and regression to the mean may have contributed to the observed changes. Second, the cohort was predominantly composed of patients with HFrEF, and the findings cannot be extrapolated to HFmrEF or HFpEF; moreover, patients were stable outpatients followed at dedicated heart failure clinics, with limited congestion (61% receiving loop diuretics), and the findings may not apply to patients initiating SGLT2i during or shortly after a heart failure hospitalisation. Third, intra- and inter-observer reproducibility of echocardiographic measurements was not formally assessed in this retrospective study, and the modest median LVEF increase lies within the range of test–retest variability of two-dimensional echocardiography [25]. Fourth, LA and RV strain analyses were available in only 51 patients, and sPAP could be estimated in a subset of patients, limiting statistical power and potentially introducing selection bias. Fifth, despite the use of penalised regression and parsimonious models, most of which were specified during the revision of the manuscript, the number of events was limited and multiple associations were tested; internal validation suggested limited optimism, but no external validation was performed; all associations should be regarded as exploratory. Sixth, the percentage of biventricular pacing in patients with CRT-D was not available, the duration of stable background therapy before SGLT2i initiation varied among patients and was not recorded, and individual changes in background therapy during the first 6 months, together with incomplete ARNI data at 6 months, may have contributed to the observed changes. Seventh, the number of clinical events was small, ventricular arrhythmias were assessed only in patients with device telemetry or Holter monitoring, which may have introduced detection bias in the broader composite endpoint, and amiodarone was used by 42% of patients, which may have influenced the occurrence of arrhythmic events. Finally, inclusion required survival and attendance at the 6-month visit, which may have introduced survivor and complete-case selection bias, and non-fatal events occurring before the 6-month visit were not systematically collected.

## 5. Conclusions

In this retrospective real-world cohort of patients with predominantly reduced LVEF, SGLT2i initiation on top of background therapy was followed at 6 months by modest reductions in LV volumes, a small increase in LVEF and a reduction in estimated pulmonary pressure. Larger baseline LV size was associated with LV volume reduction and lower baseline TAPSE with LVEF and functional response, although these associations may partly reflect regression to the mean. Given the absence of a control group, the limited sample size and the exploratory nature of the analyses, these findings should be considered hypothesis-generating and require confirmation in larger prospective controlled studies.

## Figures and Tables

**Table 1:** Baseline characteristics of the study population (n = 101).

Characteristic	Value	n
Age (years)	64 (51–71)	101
Male sex	79 (78%)	101
Height (cm)	170 (166–174)	101
Weight (kg)	80 (71–89)	101
BMI (kg/m^2^)	27.3 (24.9–30.1)	101
BSA (m^2^)	1.93 (1.83–2.04)	101
LVEF category		101
HFrEF	94 (93%)	
HFmrEF	6 (6%)	
HFpEF	1 (1%)	
Arterial hypertension	55 (54%)	101
Diabetes mellitus	17 (17%)	101
Dyslipidaemia	68 (67%)	101
Smoking (current or former)	39 (39%)	101
Atrial fibrillation	33 (33%)	101
Anaemia	29 (29%)	101
Ischaemic aetiology	37 (37%)	101
Cardiac device		101
CRT-D	19 (19%)	
ICD	50 (50%)	
None	32 (32%)	
Previous valvular intervention		51
Cardiac surgery	5 (10%)	
MitraClip/TAVI	3 (6%)	
None	43 (84%)	
Systolic BP (mmHg)	115 (110–130)	101
Diastolic BP (mmHg)	70 (70–80)	101
Heart rate (bpm)	68 (61–74)	99
NYHA class		101
I	14 (14%)	
II	58 (57%)	
III	29 (29%)	
Haemoglobin (g/dL)	13.7 (12.4–14.9)	100
Creatinine (mg/dL)	1.06 (0.90–1.20)	100
eGFR (mL/min/1.73 m^2^)	75.8 (64.0–89.5)	100
Background therapy at SGLT2i initiation		
Beta-blocker	101 (100%)	101
ARNI	67 (66%)	101
ACE inhibitor	22 (22%)	101
ARB	12 (12%)	101
MRA	80 (79%)	101
Beta-blocker + MRA + ARNI/ACEi/ARB	79 (78%)	101

Values are median (IQR) or n (%). n indicates the number of patients with available data. ACE: angiotensin-converting enzyme; ARB: angiotensin receptor blocker; ARNI: angiotensin receptor–neprilysin inhibitor; BMI: body mass index; BSA: body surface area; CRT-D: cardiac resynchronisation therapy-defibrillator; eGFR: estimated glomerular filtration rate (CKD-EPI 2021); HFmrEF/HFpEF/HFrEF: heart failure with mildly reduced/preserved/reduced ejection fraction; ICD: implantable cardioverter–defibrillator; MRA: mineralocorticoid receptor antagonist; NYHA: New York Heart Association; TAVI: transcatheter aortic valve implantation.

**Table 2:** Clinical, laboratory and echocardiographic parameters at baseline and at 6 months.

Parameter	n	Baseline	6 Months	*p*-Value
**Clinical and laboratory**				
NYHA class	101	2 (2–3)	2 (2–2)	0.696
Weight (kg)	101	80 (71–89)	78 (70–86)	0.125
BMI (kg/m^2^)	101	27.3 (24.9–30.1)	26.4 (23.8–29.8)	0.149
BSA (m^2^)	101	1.93 (1.83–2.04)	1.91 (1.81–2.04)	0.123
Systolic BP (mmHg)	101	115 (110–130)	120 (110–130)	0.872
Diastolic BP (mmHg)	101	70 (70–80)	70 (70–80)	0.803
Heart rate (bpm)	98	69 (61–74)	66 (61–72)	0.480
Haemoglobin (g/dL)	96	13.6 (12.4–14.9)	13.0 (12.0–14.4)	0.026
Creatinine (mg/dL)	96	1.06 (0.90–1.20)	1.12 (0.94–1.38)	0.048
eGFR (mL/min/1.73 m^2^)	96	75.1 (63.8–89.5)	71.4 (55.0–86.3)	0.062
**LV structure and systolic function**				
IVS (mm)	101	11 (10–12)	11 (10–13)	0.001
LVPW (mm)	101	10 (9–11)	10 (9–11)	0.113
LVEDD (mm)	101	62 (56–73)	62 (54–70)	0.021
LVEDDi (mm/m^2^)	101	33.6 (29.0–36.8)	34.6 (30.0–37.5)	0.014
LV mass (g)	101	287 (222–362)	295 (218–348)	0.001
LV mass index (g/m^2^)	101	145 (119–185)	154 (116–184)	0.560
LVEDV (mL)	101	197 (135–261)	182 (125–249)	<0.001
LVEDVi (mL/m^2^)	101	103 (71–134)	95 (68–135)	<0.001
LVESV (mL)	101	138 (91–197)	124 (76–185)	<0.001
LVESVi (mL/m^2^)	101	76 (47–101)	64 (42–92)	<0.001
LVEF (%)	101	28.8 (24.4–36.0)	31.6 (25.6–38.0)	<0.001
LV GLS (%)	99	−8.9 (−11.9–−6.7)	−9.9 (−12.4–−7.2)	0.003
**Left atrium and diastolic function**				
LAV (mL)	96	74 (55–133)	65 (51–116)	<0.001
LAVi (mL/m^2^)	95	40.2 (28.7–65.5)	36.8 (27.7–63.9)	0.104
E wave (cm/s)	89	64 (51–78)	61 (47–80)	0.035
A wave (cm/s)	79	78 (63–89)	89 (68–99)	0.001
E/A ratio	79	0.86 (0.58–1.08)	0.74 (0.52–0.89)	<0.001
Septal e′ (cm/s)	39	5 (4–6)	5 (4–6)	0.560
Lateral e′ (cm/s)	39	8 (5–10)	8 (6–10)	0.994
E/e′	89	11.4 (7.2–13.3)	11.3 (7.8–12.7)	0.466
Diastolic dysfunction grade	79	1 (1–2)	1 (1–2)	0.029
**Right ventricle and pulmonary circulation**				
RV basal diameter (mm)	51	34 (32–37)	34 (33–37)	0.546
TAPSE (mm)	95	20.3 (18.0–23.2)	21.0 (19.0–24.0)	0.135
sPAP (mmHg)	69	33 (24–40)	31 (23–35)	<0.001
TAPSE/sPAP (mm/mmHg)	68	0.78 (0.54–0.85)	0.79 (0.61–0.90)	<0.001
**Valvular regurgitation (grade 0–3)**				
Mitral regurgitation	101	1 (1–2)	1 (1–2)	0.618
Aortic regurgitation	101	0 (0–1)	0 (0–1)	0.903
Tricuspid regurgitation	93	1 (1–1)	1 (1–2)	0.814

Values are median (IQR) at each time point in patients with paired data (complete cases for each variable pair); they are not medians of individual changes, which are reported for LV volumes and LVEF ; n is the number of patients contributing to each comparison. Wilcoxon signed-rank test. A: late diastolic transmitral velocity; BSA: body surface area; E: early diastolic transmitral velocity; e′: early diastolic mitral annular velocity; GLS: global longitudinal strain; IVS: interventricular septum; LAV(i): left atrial volume (index); LVEDD(i): LV end-diastolic diameter (index); LVEDV(i)/LVESV(i): LV end-diastolic/end-systolic volume (index); LVPW: LV posterior wall; RV: right ventricle; sPAP: systolic pulmonary artery pressure; TAPSE: tricuspid annular plane systolic excursion.

**Table 3:** Left atrial and right ventricular strain at baseline and at 6 months (subgroup with available analysis, n = 51).

Parameter	n	Baseline	6 Months	*p*-Value
LA reservoir strain (%)	51	17.7 (13.2–25.5)	23.6 (14.7–28.1)	0.173
RV free-wall strain (%)	51	−19.5 (−23.8–−15.3)	−21.0 (−24.2–−17.2)	0.223
RV global longitudinal strain (%)	51	−14.9 (−18.4–−12.1)	−14.8 (−18.1–−12.6)	0.873

Values are median (IQR). Wilcoxon signed-rank test. LV GLS, available in 99 patients, is reported in Table 2.

**Table 4:** Changes in LV volumes and LVEF and proportion of patients meeting each response definition (n = 101).

Parameter/Definition	n	Median (IQR) or n (%)	Mean (95% CI)
**Change from baseline to 6 months**			
ΔLVEDV (mL)	101	−28.0 (−48.6 to −2.2)	−20.8 (−33.3 to −8.4)
ΔLVEDV (%)	101	−13.0 (−20.9 to −0.9)	−1.2 (−10.7 to 8.3)
ΔLVESV (mL)	101	−24.0 (−42.0 to −5.5)	−19.7 (−29.9 to −9.5)
ΔLVESV (%)	101	−16.3 (−24.7 to −4.8)	−1.6 (−13.5 to 10.4)
ΔLVEF (percentage points)	101	2.3 (1.0 to 4.8)	2.7 (0.9 to 4.6)
**Response definitions**			
Operational LV reverse remodelling (any reduction in both LVEDV and LVESV)	101	74 (73.3%)	
LVESV reduction ≥ 10%	101	69 (68.3%)	
LVESV reduction ≥ 15%	101	56 (55.4%)	
LVEF increase ≥ 5 percentage points	101	24 (23.8%)	
NYHA improvement ≥ 1 class	101	23 (22.8%)	
Combined echocardiographic and functional response	101	8 (7.9%)	

Changes are individual paired changes (6-month minus baseline value), summarised as median (IQR) and mean (95% CI); ΔLVEDV and ΔLVESV are expressed in mL and as percentage of the baseline value; negative values indicate a reduction. LVEF: left ventricular ejection fraction; LVEDV/LVESV: LV end-diastolic/end-systolic volume; NYHA: New York Heart Association.

**Table 5:** Baseline characteristics associated with the operational definition of LV reverse remodelling (simultaneous reduction in LVEDV and LVESV): Firth penalised logistic regression.

Variable	Increment	Unadjusted OR (95% CI)	*p*	Adjusted OR (95% CI) †	*p*	n (Events)
Age	per 10 years	0.79 (0.56–1.10)	0.166	0.88 (0.61–1.26) ‡	0.476	101 (74)
Male sex	yes vs. no	1.40 (0.50–3.91)	0.516	1.56 (0.51–4.73)	0.432	101 (74)
BMI	per 1 kg/m^2^	1.27 (1.09–1.48)	0.003	1.30 (1.09–1.56)	0.003	101 (74)
Arterial hypertension	yes vs. no	0.95 (0.39–2.29)	0.903	1.41 (0.51–3.87)	0.509	101 (74)
Diabetes mellitus	yes vs. no	0.82 (0.26–2.56)	0.730	1.01 (0.28–3.71)	0.984	101 (74)
Atrial fibrillation	yes vs. no	1.18 (0.46–3.05)	0.730	1.48 (0.51–4.32)	0.471	101 (74)
Anaemia	yes vs. no	2.69 (0.87–8.35)	0.087	2.19 (0.66–7.22)	0.199	101 (74)
Ischaemic aetiology	yes vs. no	0.96 (0.39–2.39)	0.939	1.29 (0.46–3.60)	0.622	101 (74)
NYHA class	per 1 class	0.89 (0.44–1.78)	0.739	0.39 (0.16–0.98)	0.045	101 (74)
Systolic BP	per 10 mmHg	0.89 (0.68–1.17)	0.413	1.00 (0.74–1.36)	0.992	101 (74)
Heart rate	per 10 bpm	0.83 (0.58–1.19)	0.313	0.78 (0.51–1.19)	0.243	99 (72)
Haemoglobin	per 1 g/dL	0.82 (0.61–1.09)	0.163	0.80 (0.59–1.10)	0.176	100 (74)
eGFR	per 10 mL/min/1.73 m^2^	0.96 (0.78–1.17)	0.667	0.82 (0.62–1.07)	0.137	100 (74)
LVEF	per 5%	0.52 (0.36–0.74)	<0.001	0.53 (0.37–0.76) ‡	<0.001	101 (74)
LVEDV	per 10 mL	1.35 (1.18–1.54)	<0.001	1.37 (1.18–1.60)	<0.001	101 (74)
LAV	per 10 mL	1.25 (1.09–1.44)	0.001	1.23 (1.06–1.43)	0.005	96 (69)
E/e′	per 1 unit	1.00 (0.90–1.11)	0.959	0.94 (0.84–1.05)	0.275	95 (70)
Diastolic dysfunction grade	per 1 grade	1.82 (0.74–4.46)	0.193	1.26 (0.51–3.15)	0.614	88 (69)
TAPSE	per 1 mm	0.97 (0.86–1.08)	0.534	0.92 (0.80–1.05)	0.218	99 (73)
sPAP	per 5 mmHg	0.81 (0.63–1.04)	0.097	0.76 (0.59–0.98)	0.032	83 (66)
LV GLS magnitude (|GLS|)	per 1% (higher = better)	0.82 (0.71–0.95)	0.007	0.94 (0.76–1.16)	0.563	99 (72)
Mitral regurgitation grade	per 1 grade	1.03 (0.60–1.79)	0.907	0.91 (0.50–1.69)	0.775	101 (74)
CRT-D	yes vs. no	1.97 (0.55–7.07)	0.296	1.18 (0.27–5.08)	0.829	101 (74)
ICD	yes vs. no	0.72 (0.30–1.75)	0.474	0.46 (0.17–1.29)	0.140	101 (74)

Each characteristic was entered unadjusted and in a model adjusted for age and baseline LVEF († ‡ adjusted for the other core covariate only). |GLS|: absolute value of LV global longitudinal strain (higher values indicate better function). n (events) refers to the adjusted model. Abbreviations as in Table 1 and Table 2.Abbreviations: n indicates the number of patients with available data; ACE: angiotensin-converting enzyme; ARB: angiotensin receptor blocker; ARNI: angiotensin receptor–neprilysin inhibitor; BMI: body mass index; BSA: body surface area; CRT-D: cardiac resynchronisation therapy-defibrillator; eGFR: estimated glomerular filtration rate; HFmrEF/HFpEF/HFrEF: heart failure with mildly reduced/preserved/reduced ejection fraction; ICD: implantable cardioverter-defibrillator; MRA: mineralocorticoid receptor antagonist; NYHA: New York Heart Association; TAVI: transcatheter aortic valve implantation; A: late diastolic transmitral velocity; BSA: body surface area; E: early diastolic transmitral velocity; e′: early diastolic mitral annular velocity; GLS: global longitudinal strain; IVS: interventricular septum; LAV(i): left atrial volume (index); LVEDD(i): LV end-diastolic diameter (index); LVEDV(i)/LVESV(i): LV end-diastolic/end-systolic volume (index); LVPW: LV posterior wall; RV: right ventricle; sPAP: systolic pulmonary artery pressure; TAPSE: tricuspid annular plane systolic excursion.

**Table 6:** Baseline characteristics associated with LVEF response (ΔLVEF ≥5 percentage points): Firth penalised logistic regression.

Variable	Increment	Unadjusted OR (95% CI)	*p*	Adjusted OR (95% CI) †	*p*	n (Events)
Age	per 10 years	0.98 (0.71–1.36)	0.924	1.02 (0.73–1.43) ‡	0.900	101 (24)
Male sex	yes vs. no	0.44 (0.16–1.23)	0.119	0.44 (0.16–1.24)	0.120	101 (24)
BMI	per 1 kg/m^2^	0.88 (0.76–1.01)	0.066	0.87 (0.75–1.00)	0.054	101 (24)
Arterial hypertension	yes vs. no	0.79 (0.32–1.97)	0.618	0.82 (0.32–2.13)	0.686	101 (24)
Diabetes mellitus	yes vs. no	0.24 (0.04–1.42)	0.115	0.22 (0.04–1.33)	0.099	101 (24)
Atrial fibrillation	yes vs. no	1.06 (0.40–2.78)	0.905	1.08 (0.41–2.83)	0.880	101 (24)
Anaemia	yes vs. no	2.17 (0.83–5.66)	0.112	1.99 (0.76–5.25)	0.163	101 (24)
Ischaemic aetiology	yes vs. no	1.06 (0.42–2.73)	0.896	1.09 (0.41–2.89)	0.860	101 (24)
NYHA class	per 1 class	0.93 (0.45–1.89)	0.834	0.74 (0.34–1.63)	0.460	101 (24)
Systolic BP	per 10 mmHg	0.69 (0.50–0.95)	0.024	0.67 (0.47–0.95)	0.023	101 (24)
Heart rate	per 10 bpm	1.06 (0.72–1.56)	0.773	1.05 (0.70–1.56)	0.828	99 (22)
Haemoglobin	per 1 g/dL	0.88 (0.66–1.16)	0.363	0.88 (0.66–1.17)	0.385	100 (24)
eGFR	per 10 mL/min/1.73 m^2^	1.03 (0.84–1.26)	0.805	1.03 (0.81–1.30)	0.832	100 (24)
LVEF	per 5%	0.82 (0.58–1.17)	0.275	0.82 (0.58–1.17) ‡	0.275	101 (24)
LVEDV	per 10 mL	0.94 (0.88–1.00)	0.037	0.82 (0.74–0.91)	<0.001	101 (24)
LAV	per 10 mL	0.87 (0.76–0.98)	0.026	0.85 (0.74–0.96)	0.012	96 (24)
E/e′	per 1 unit	1.06 (0.96–1.18)	0.248	1.05 (0.94–1.17)	0.365	95 (22)
Diastolic dysfunction grade	per 1 grade	1.28 (0.60–2.77)	0.524	1.22 (0.55–2.70)	0.630	88 (21)
TAPSE	per 1 mm	0.72 (0.61–0.85)	<0.001	0.66 (0.53–0.81)	<0.001	99 (22)
sPAP	per 5 mmHg	1.32 (1.01–1.72)	0.039	1.31 (1.01–1.70)	0.043	83 (16)
LV GLS magnitude (|GLS|)	per 1% (higher = better)	1.10 (0.96–1.27)	0.178	1.47 (1.15–1.87)	0.002	99 (24)
Mitral regurgitation grade	per 1 grade	1.26 (0.72–2.22)	0.421	1.21 (0.68–2.16)	0.514	101 (24)
CRT-D	yes vs. no	1.68 (0.56–5.00)	0.352	1.39 (0.42–4.52)	0.589	101 (24)
ICD	yes vs. no	0.54 (0.21–1.37)	0.193	0.50 (0.19–1.28)	0.147	101 (24)

† Adjusted for age and baseline LVEF; ‡ adjusted for the other core covariate only. Abbreviations as in Table 1, Table 2 and Table 5.

**Table 7:** Baseline characteristics associated with NYHA class improvement (≥1 class): Firth penalised logistic regression.

Variable	Increment	Unadjusted OR (95% CI)	*p*	Adjusted OR (95% CI) †	*p*	n (Events)
Age	per 10 years	1.17 (0.82–1.65)	0.388	1.28 (0.88–1.85) ‡	0.197	101 (23)
Male sex	yes vs. no	1.92 (0.54–6.82)	0.314	1.90 (0.52–6.91)	0.329	101 (23)
BMI	per 1 kg/m^2^	1.03 (0.90–1.18)	0.672	1.02 (0.89–1.18)	0.773	101 (23)
Arterial hypertension	yes vs. no	1.38 (0.54–3.53)	0.502	1.37 (0.51–3.70)	0.538	101 (23)
Diabetes mellitus	yes vs. no	1.12 (0.33–3.75)	0.855	0.91 (0.25–3.31)	0.890	101 (23)
Atrial fibrillation	yes vs. no	1.46 (0.56–3.80)	0.441	1.49 (0.56–3.99)	0.423	101 (23)
Anaemia	yes vs. no	1.14 (0.42–3.12)	0.797	0.91 (0.32–2.57)	0.852	101 (23)
Ischaemic aetiology	yes vs. no	1.46 (0.57–3.73)	0.433	1.36 (0.51–3.65)	0.541	101 (23)
NYHA class	per 1 class	7.01 (2.69–18.30)	<0.001	5.97 (2.12–16.78)	<0.001	101 (23)
Systolic BP	per 10 mmHg	0.96 (0.72–1.27)	0.760	0.94 (0.68–1.28)	0.682	101 (23)
Heart rate	per 10 bpm	1.25 (0.86–1.80)	0.237	1.21 (0.82–1.78)	0.330	99 (23)
Haemoglobin	per 1 g/dL	1.02 (0.76–1.37)	0.895	1.06 (0.78–1.45)	0.719	100 (22)
eGFR	per 10 mL/min/1.73 m^2^	0.99 (0.80–1.22)	0.897	1.02 (0.80–1.31)	0.858	100 (22)
LVEF	per 5%	0.69 (0.47–1.01)	0.059	0.65 (0.44–0.98) ‡	0.038	101 (23)
LVEDV	per 10 mL	1.01 (0.95–1.07)	0.811	0.94 (0.86–1.02)	0.122	101 (23)
LAV	per 10 mL	0.96 (0.85–1.07)	0.439	0.92 (0.82–1.03)	0.160	96 (23)
E/e′	per 1 unit	1.17 (1.03–1.32)	0.015	1.12 (0.99–1.27)	0.071	95 (23)
Diastolic dysfunction grade	per 1 grade	1.28 (0.60–2.77)	0.524	1.00 (0.43–2.33)	0.996	88 (21)
TAPSE	per 1 mm	0.87 (0.77–0.99)	0.029	0.82 (0.71–0.95)	0.009	99 (22)
sPAP	per 5 mmHg	1.40 (1.07–1.82)	0.013	1.38 (1.04–1.83)	0.024	83 (19)
LV GLS magnitude (|GLS|)	per 1% (higher = better)	0.91 (0.78–1.05)	0.189	1.07 (0.85–1.33)	0.577	99 (23)
Mitral regurgitation grade	per 1 grade	1.85 (1.03–3.32)	0.041	1.63 (0.89–2.98)	0.112	101 (23)
CRT-D	yes vs. no	1.32 (0.43–4.11)	0.629	0.72 (0.20–2.50)	0.601	101 (23)
ICD	yes vs. no	1.42 (0.56–3.60)	0.457	1.34 (0.51–3.49)	0.550	101 (23)

† Adjusted for age and baseline LVEF; ‡ adjusted for the other core covariate only. Abbreviations as in Table 1, Table 2 and Table 5.

**Table 8:** Clinical events after the 6-month visit and association between 6-month changes and death or heart failure hospitalisation.

*(A) Clinical events after the 6-month visit.*							
**Event**						**n (%)**	
Follow-up after the 6-month visit, months, median (IQR)						32 (25–33)	
Death or HF hospitalisation						30 (29.7%)	
All-cause death						14 (13.9%)	
HF hospitalisation						18 (17.8%)	
**Other events (descriptive)**							
Non-fatal MI/PCI						2 (2.0%)	
Incident atrial fibrillation §						14/68 (20.6%)	
Ventricular arrhythmias (NSVT/VT/VF) †						14/55 (25.5%)	
*(B) Association between 6-month changes and death or HF hospitalisation (adjusted for age and baseline LVEF).*							
**Change at 6 months**	**Increment**	**Death or HFH HR (95% CI)**	***p***	**Death HR (95% CI)**	***p***	**HFH HR (95% CI)**	***p***
Change in LVEF	per 5-point increase	0.87 (0.68–1.12)	0.293	0.64 (0.43–0.94)	0.024	1.00 (0.74–1.35)	0.997
Relative change in LVESV	per 10% reduction	1.00 (0.92–1.09)	0.930	1.01 (0.89–1.15)	0.849	1.02 (0.92–1.13)	0.730
Relative change in LVEDV	per 10% reduction	1.01 (0.92–1.12)	0.798	1.07 (0.92–1.25)	0.363	1.01 (0.90–1.14)	0.811
LVEF increase ≥ 5 points	yes vs. no	1.51 (0.66–3.48)	0.331	0.63 (0.14–2.86)	0.547	2.53 (0.89–7.20)	0.083
LVESV reduction ≥ 15%	yes vs. no	0.88 (0.42–1.84)	0.728	1.04 (0.35–3.11)	0.950	0.68 (0.26–1.76)	0.425
Operational LV reverse remodelling	yes vs. no	0.95 (0.37–2.39)	0.907	0.89 (0.23–3.42)	0.866	0.89 (0.28–2.81)	0.846
NYHA improvement ≥ 1 class	yes vs. no	1.46 (0.63–3.38)	0.372	n.e. ‡	0.046	4.20 (1.53–11.52)	0.005

Time origin: 6-month visit (landmark). (B) Cox regression adjusted for age and baseline LVEF; 30 events for death or heart failure hospitalisation, 14 for all-cause death and 18 for heart failure hospitalisation. Relative changes in LVESV and LVEDV were analysed as the log-transformed ratio of the 6-month to the baseline value. † Assessed only in patients with available device telemetry or Holter monitoring (n = 55). § Patients without a history of AF (n = 68). ‡ Not estimable: no deaths among patients with NYHA improvement (log-rank *p* shown). HFH: heart failure hospitalisation; MI: myocardial infarction; NSVT: non-sustained ventricular tachycardia; PCI: percutaneous coronary intervention; VF: ventricular fibrillation; VT: ventricular tachycardia; other abbreviations as in Table 1 and Table 2.

**Table 9:** Background pharmacological therapy at baseline and at 6 months.

***(A) Drug utilisation.***						
**Drug**	**Baseline**	**6 Months**	**n Paired**	**Started**	**Stopped**	***p*** **-Value**
Loop diuretic (furosemide)	62/101 (61.4%)	57/101 (56.4%)	101	12	17	0.353
MRA	80/101 (79.2%)	83/101 (82.2%)	101	12	9	0.513
Beta-blocker	101/101 (100.0%)	101/101 (100.0%)	101	0	0	n.a.
ACE inhibitor	22/101 (21.8%)	23/101 (22.8%)	101	12	11	0.835
ARB	12/101 (11.9%)	13/101 (12.9%)	101	6	5	0.763
ARNI	67/101 (66.3%)	36/68 (52.9%)	68	15	18	0.602
GLP-1 RA	0/101 (0.0%)	1/101 (1.0%)	101	1	0	0.317
Digoxin	4/51 (7.8%)	4/51 (7.8%)	51	3	3	1.000
Statin	67/101 (66.3%)	68/101 (67.3%)	101	11	10	0.827
Ezetimibe	34/101 (33.7%)	37/101 (36.6%)	101	9	6	0.439
Aspirin	39/101 (38.6%)	40/101 (39.6%)	101	14	13	0.847
DOAC	29/101 (28.7%)	34/100 (34.0%)	100	18	13	0.369
VKA	8/51 (15.7%)	8/51 (15.7%)	51	7	7	1.000
Metformin	6/101 (5.9%)	7/101 (6.9%)	101	3	2	0.655
Insulin	3/101 (3.0%)	3/101 (3.0%)	101	1	1	1.000
Amiodarone	42/101 (41.6%)	47/101 (46.5%)	101	16	11	0.336
*(B) Doses.*						
**Drug**		**n**	**Baseline**	**6 months**		***p*** **-value**
Furosemide (mg/day)		44	43.8 (25.0–81.2)	43.8 (25.0–75.0)		0.975
MRA (mg/day)		71	50.0 (25.0–50.0)	50.0 (25.0–50.0)		0.763
Beta-blocker (% of target dose)		76	25 (12–50)	38 (12–75)		0.097
Sacubitril/valsartan (mg/day, as recorded)		17	50 (50–50)	50 (50–100)		1.000

Values are n/N (%), where N is the number of patients with available data at each time point. McNemar’s test in patients with data at both time points; Started/Stopped: number of patients starting or discontinuing the drug between baseline and 6 months. Doses: median (IQR) in patients receiving the drug at both time points (β-blocker: same molecule at both time points; target doses: metoprolol 200 mg, bisoprolol 10 mg, carvedilol 50 mg daily); Wilcoxon signed-rank test. DOAC: direct oral anticoagulant; GLP-1 RA: glucagon-like peptide-1 receptor agonist; MRA: mineralocorticoid receptor antagonist; VKA: vitamin K antagonist; other abbreviations as in Table 1.

## Data Availability

The data presented in this study are not publicly available due to privacy restrictions.

## Supplementary Materials

The following supporting information can be downloaded at https://www.mdpi.com/article/10.3390/jcm15218679/s1. Figure S1: Spearman correlation matrix of baseline continuous characteristics. Figure S2: Event-free survival for the broader composite endpoint according to LVEF response. Figure S3: Event-free survival for the broader composite endpoint according to NYHA class change. Figure S4: Event-free survival for the broader composite endpoint according to the operational definition of LV reverse remodelling. Table S1: Available and missing data for each paired comparison. Table S2: Events per parameter and variance inflation factors of the original and revised modelling strategies. Table S3: Tests of departure from linearity. Table S4: Bootstrap stability of key associations. Table S5: Internal validation of the main logistic and Cox models. Table S6: Sensitivity analyses in patients with HFrEF and after exclusion of patients with CRT-D. Table S7: Baseline characteristics associated with LVESV reduction ≥15%. Table S8: Baseline characteristics associated with LVESV reduction ≥10%. Table S9: ANCOVA models of 6-month LVESV, LVEDV and LVEF. Table S10: Baseline characteristics associated with combined echocardiographic and functional response. Table S11: Baseline characteristics and death or heart failure hospitalisation. Table S12: Broader composite endpoint: events and Cox regression. Table S13: Multivariable Cox models for death or heart failure hospitalisation and the broader composite endpoints. Table S14: Association between baseline LVEF and the individual components of the broader composite endpoint. Table S15: Association between 6-month response and the broader composite endpoint. Table S16: Association between 6-month response and the broader composite endpoint excluding ventricular arrhythmias. Table S17: LV changes in patients without documented changes in background disease-modifying therapy.

## Abbreviations

The following abbreviations are used in this manuscript:

ACE, angiotensin-converting enzyme; ANCOVA, analysis of covariance; ARB, angiotensin receptor blocker; AF, atrial fibrillation; ARNI, angiotensin receptor–neprilysin inhibitor; BMI, body mass index; BSA, body surface area; CABG, coronary artery bypass grafting; CI, confidence interval; CKD-EPI, Chronic Kidney Disease Epidemiology Collaboration; CRT(-D), cardiac resynchronisation therapy(-defibrillator); DOAC, direct oral anticoagulant; eGFR, estimated glomerular filtration rate; GLP-1 RA, glucagon-like peptide-1 receptor agonist; GLS, global longitudinal strain; HF, heart failure; HFmrEF, heart failure with mildly reduced ejection fraction; HFpEF, heart failure with preserved ejection fraction; HFrEF, heart failure with reduced ejection fraction; HR, hazard ratio; ICD, implantable cardioverter–defibrillator; IQR, interquartile range; IVS, interventricular septum; LA, left atrial; LARS, left atrial reservoir strain; LAV(i), left atrial volume (index); LV, left ventricular; LVEDD(i), left ventricular end-diastolic diameter (index); LVEDV(i), left ventricular end-diastolic volume (index); LVEF, left ventricular ejection fraction; LVESV(i), left ventricular end-systolic volume (index); LVPW, left ventricular posterior wall; MI, myocardial infarction; MRA, mineralocorticoid receptor antagonist; NSVT, non-sustained ventricular tachycardia; NYHA, New York Heart Association; OR, odds ratio; PCI, percutaneous coronary intervention; RV, right ventricular; RVFWS, right ventricular free-wall strain; RVGLS, right ventricular global longitudinal strain; SGLT2i, sodium–glucose cotransporter-2 inhibitors; sPAP, systolic pulmonary arterial pressure; TAPSE, tricuspid annular plane systolic excursion; TAVI, transcatheter aortic valve implantation; VF, ventricular fibrillation; VIF, variance inflation factor; VKA, vitamin K antagonist; VT, ventricular tachycardia.
