# Supplementary material for: Changes in Left Ventricular Volumes and Function After Initiation of Sodium–Glucose Cotransporter-2 Inhibitors in Patients with Heart Failure and Predominantly Reduced Ejection Fraction: A Real-World Echocardiographic Study
Source: J Clin Med. 2026 Nov 6;15(21):8679. doi: 10.3390/jcm15218679 (PMC13763686; doi:10.3390/jcm15218679)
Supplement: Supplementary file 1 [file jcm-15-08679-s001.zip › jcm-4581909-supplementary.pdf]

Supplementary Materials

Changes in Left Ventricular Volumes and Function after Initiation of Sodium–Glucose Cotransporter-2 Inhibitors in Patients with Heart Failure and Predominantly Reduced Ejection Fraction: A Real-World Echocardiographic Study

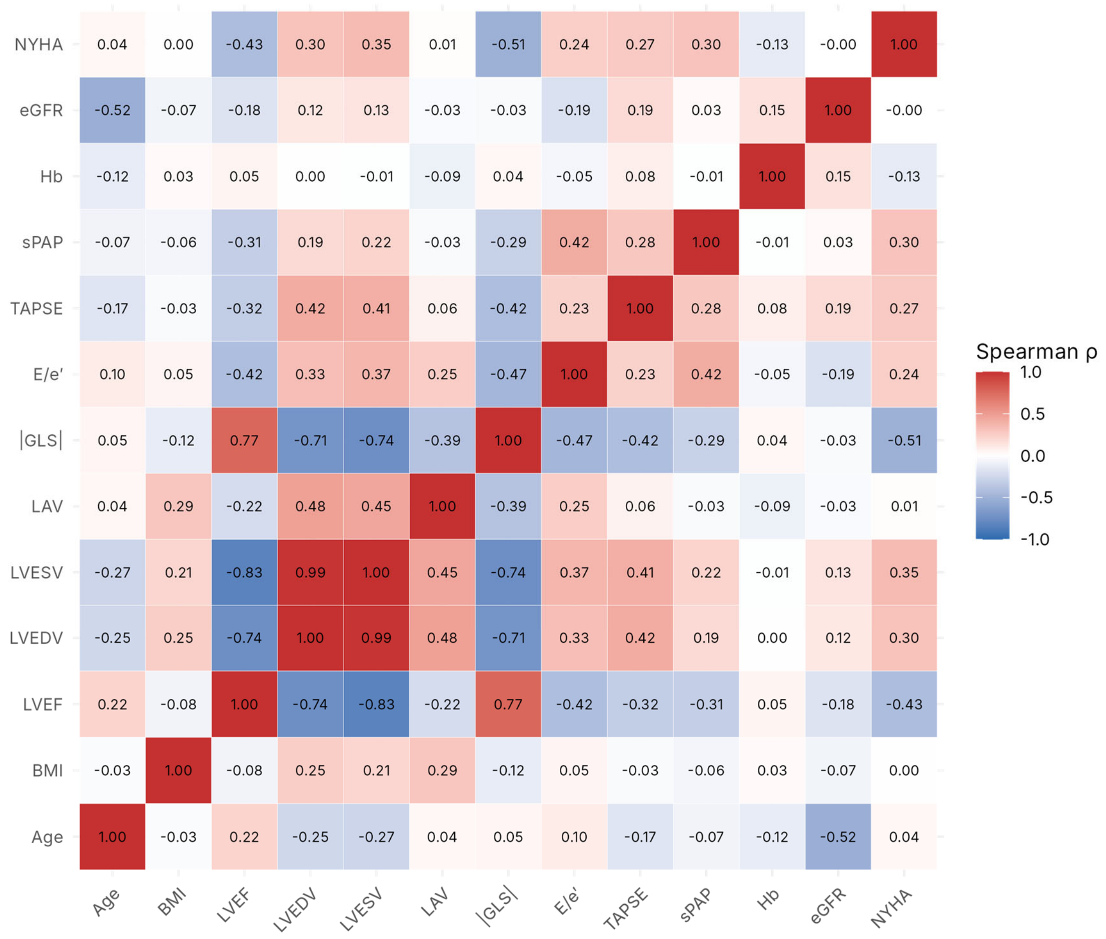

Figure S1. Spearman correlation matrix of baseline continuous characteristics.

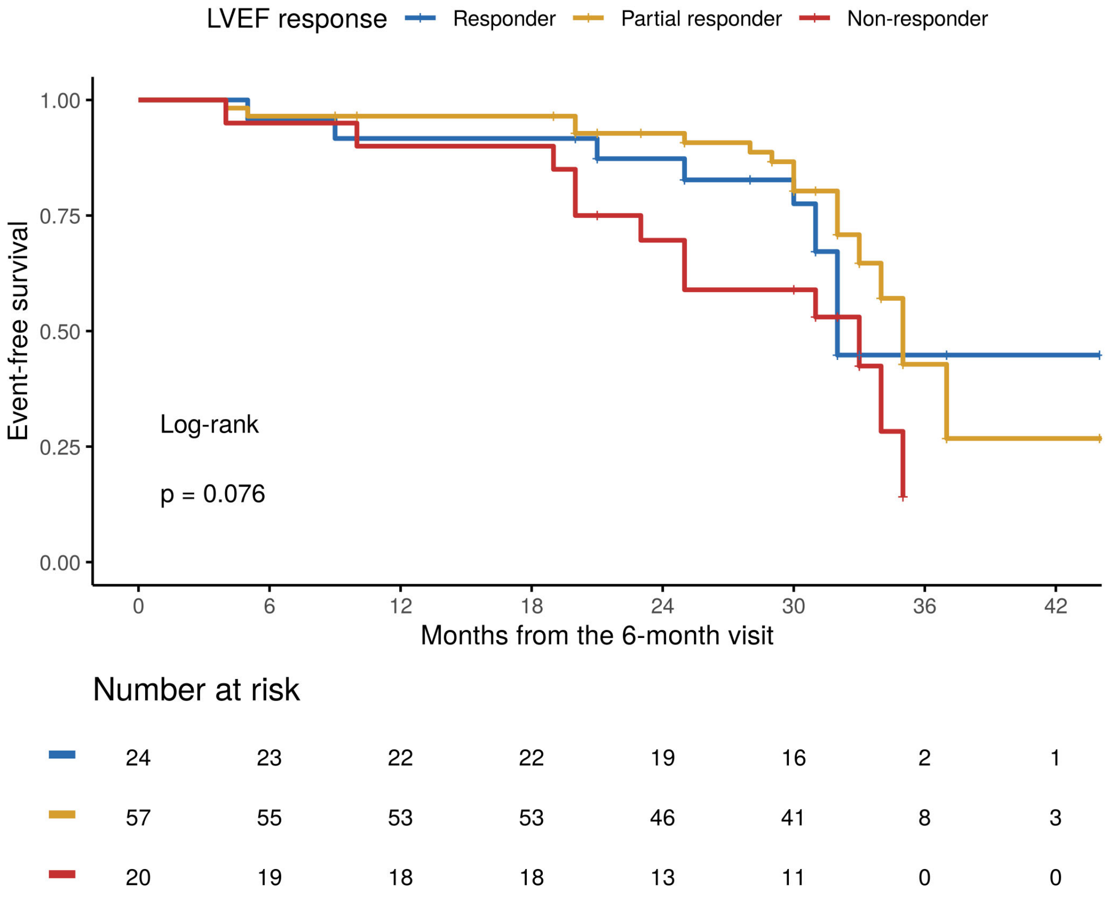

Figure S2. Event-free survival for the broader composite endpoint according to LVEF response at 6 months (responder: ΔLVEF ≥5 percentage points; partial responder: 0–4.9; non-responder: <0) (Kaplan–Meier, time origin at the 6-month visit; log-rank test; numbers at risk shown).

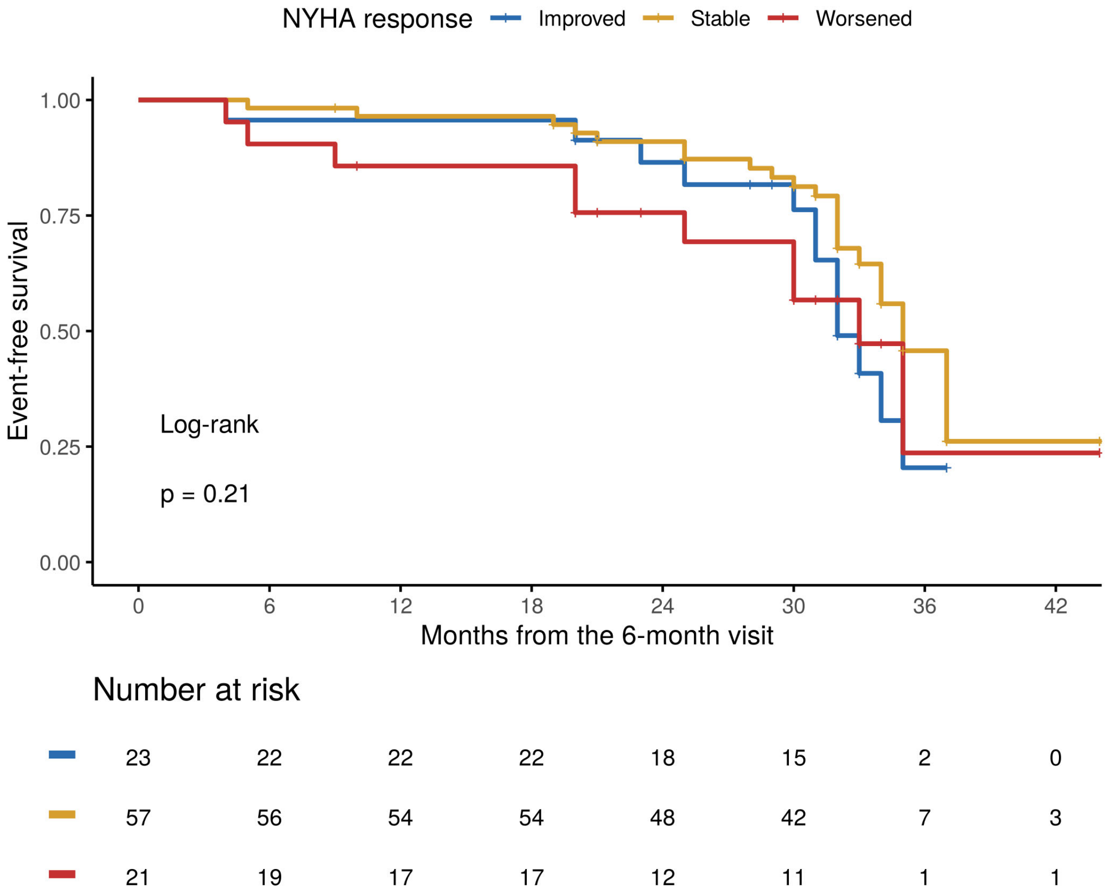

Figure S3. Event-free survival for the broader composite endpoint according to NYHA class change at 6 months (improved: ≥1 class; stable; worsened) (Kaplan–Meier, time origin at the 6-month visit; log-rank test; numbers at risk shown).

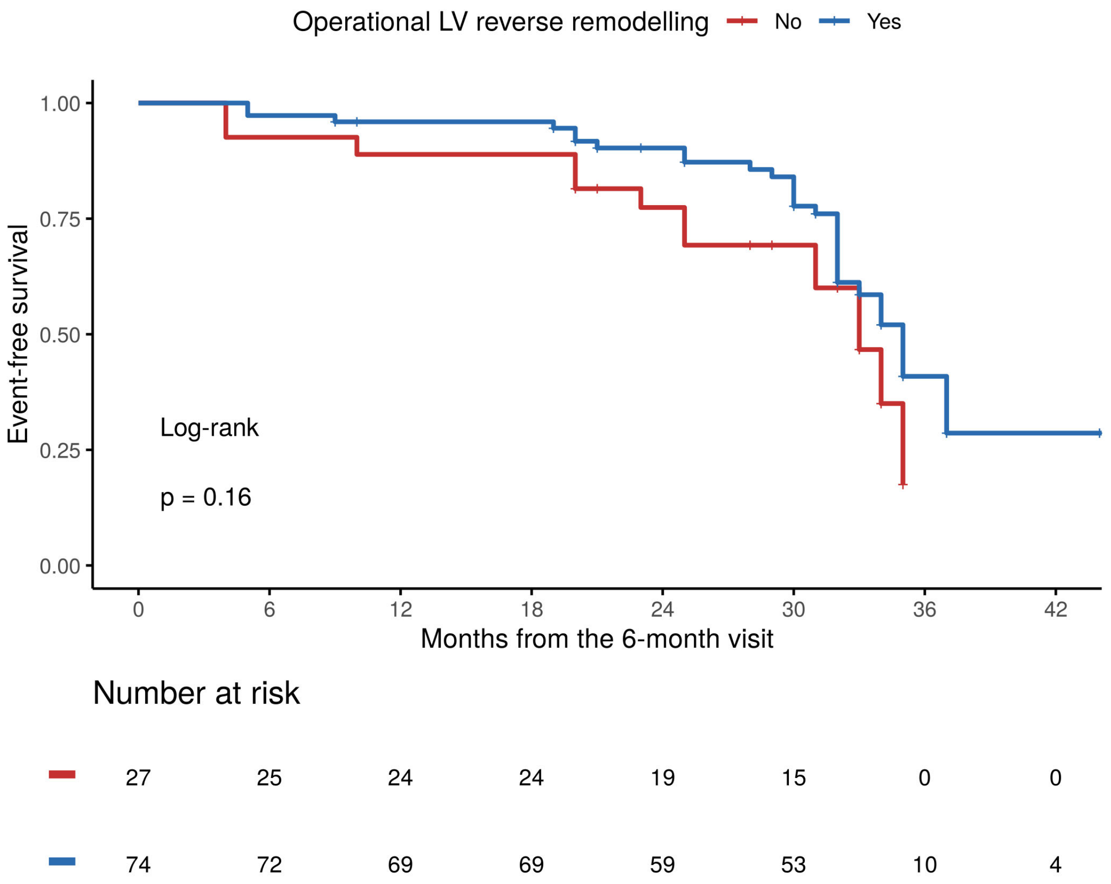

Figure S4. Event-free survival for the broader composite endpoint according to the operational definition of LV reverse remodelling (Kaplan–Meier, time origin at the 6-month visit).

Table S1. Available and missing data for each paired comparison (complete-case analysis).

Variable	Available at baseline	Available at follow-up	Paired (complete cases)	Missing paired (%)
LVEDV	101	101	101	0%
LVESV	101	101	101	0%
LVEF	101	101	101	0%
LV GLS	99	101	99	2%
LAV	96	101	96	5%
LAVi	97	95	95	6%
E wave	95	95	89	12%
A wave	88	90	79	22%
Septal/lateral e′	95	45	39	61%
E/e′	95	95	89	12%
Diastolic dysfunction grade	88	90	79	22%
RV basal diameter	101	51	51	50%
TAPSE	99	97	95	6%
sPAP	83	83	69	32%
TAPSE/sPAP	82	82	68	33%
LA reservoir strain	51	51	51	50%
RV free-wall strain	51	51	51	50%
RV GLS	51	51	51	50%
Haemoglobin	100	97	96	5%

Variable	Available at baseline	Available at follow-up	Paired (complete cases)	Missing paired (%)
Creatinine/eGFR	100	97	96	5%
Heart rate	99	100	98	3%
Tricuspid regurgitation	95	93	93	8%

Table S2. Events per estimated parameter (EPV) and maximum variance inflation factor (VIF) of the original multivariable models (univariable screening at p<0.10, simultaneous entry) and of the revised models (characteristic + age + baseline LVEF). The maximum VIF across all revised models was ≤2.92.

Endpoint	Original: n	Original: limiting events	Original: parameters	Original: EPV	Original: max VIF (variable)	Revised: limiting events	Revised: parameters	Revised: EPV
LVEF increase ≥5 points	77	15	7	2.1	1.51 (TAPSE)	24	3	8
NYHA improvement ≥1 class	78	18	6	3	4.69 (TAPSE)	23	3	7.7
Operational LV reverse remodelling	76	17	7	2.4	10.94 (GLS)	27	3	9

Table S3. Tests of departure from linearity (likelihood-ratio test comparing a natural cubic spline with 3 degrees of freedom vs. a linear term, models adjusted for age and baseline LVEF). Values are p-values.

Variable	Operational LV reverse remodelling	LVESV reduction ≥15%	LVEF increase ≥5 points	NYHA improvement
Age	0.655	0.605	0.875	0.084
BMI	0.993	0.998	0.893	0.492
LVEF	0.340	0.043	0.032	0.032
LVEDV	0.060	0.012	0.479	0.002
LAV	0.447	0.983	0.555	0.560
ǀGLSǀ	0.052	0.047	0.006	0.017
TAPSE	0.458	0.058	0.762	0.007
sPAP	0.618	0.577	0.920	0.286
E/e′	0.093	0.153	0.065	0.009
Haemoglobin	0.962	0.961	0.920	0.453

Table S4. Bootstrap stability (1000 resamples) of the associations of key characteristics in Firth logistic models adjusted for age and baseline LVEF.

Variable	Bootstrap 95% CI of adjusted OR	Same direction as original estimate (%)	p<0.05 (%)
**Operational LV reverse remodelling**			
BMI	1.13–1.65	100	92
Anaemia	0.78–10.71	94	18
LVEDV	1.24–1.86	100	100
LV GLS magnitude (ǀGLSǀ)	0.74–1.19	74	8
TAPSE	0.80–1.03	94	15
sPAP	0.55–0.94	99	61
NYHA class	0.12–1.00	97	53
LVEF	0.36–0.71	100	97
**LVESV reduction ≥15%**			
BMI	1.18–1.64	100	99
Anaemia	0.69–4.50	88	17
LVEDV	1.02–1.20	99	61

Variable	Bootstrap 95% CI of adjusted OR	Same direction as original estimate (%)	p<0.05 (%)
LV GLS magnitude (∣GLS∣)	1.02–1.55	98	40
TAPSE	0.70–0.89	100	96
sPAP	0.60–1.01	96	36
NYHA class	0.27–1.09	96	33
LVEF	0.65–1.11	88	18
**LVEF increase ≥5 points**			
BMI	0.71–1.00	98	50
Anaemia	0.73–5.34	91	27
LVEDV	0.71–0.89	100	99
LV GLS magnitude (∣GLS∣)	1.18–2.24	100	91
TAPSE	0.48–0.75	100	100
sPAP	1.01–1.95	98	51
NYHA class	0.30–1.87	76	10
LVEF	0.55–1.11	90	14
**NYHA improvement ≥1 class**			
BMI	0.88–1.18	61	3
Anaemia	0.24–2.58	59	5
LVEDV	0.83–1.02	94	34
LV GLS magnitude (∣GLS∣)	0.88–1.43	74	7
TAPSE	0.66–0.93	100	79
sPAP	1.09–2.32	100	69
NYHA class	2.21–24.15	100	94
LVEF	0.43–0.88	100	58

Table S5. Internal validation of the main models (500 bootstrap resamples). Logistic models: Firth penalised regression including the characteristic, age and baseline LVEF; Cox models: age, baseline LVEF and NYHA class (time origin: 6-month visit). Optimism is the mean difference between bootstrap and test performance; the optimism-corrected calibration slope is the slope of the bootstrap linear predictor applied to the original sample (ideal value 1).

Endpoint	Model	n (events)	Apparent C	Optimism	Optimism-corrected C	Corrected calibration slope
Operational LV reverse remodelling	BMI + age + LVEF	101 (74)	0.81	0.017	0.79	1.01
Operational LV reverse remodelling	LVEDV + age + LVEF	101 (74)	0.90	0.014	0.89	0.98
LVESV reduction ≥15%	BMI + age + LVEF	101 (56)	0.75	0.022	0.73	0.97
LVESV reduction ≥15%	LVEDV + age + LVEF	101 (56)	0.64	0.040	0.60	0.85
LVESV reduction ≥15%	TAPSE + age + LVEF	99 (55)	0.70	0.030	0.67	0.93
LVEF increase ≥5 points	TAPSE + age + LVEF	99 (22)	0.83	0.026	0.80	0.96
LVEF increase ≥5 points	sPAP + age + LVEF	83 (16)	0.69	0.064	0.63	0.81
LVEF increase ≥5 points	LV GLS magnitude (∣GLS∣) + age + LVEF	99 (24)	0.75	0.035	0.72	0.92
LVEF increase ≥5 points	LVEDV + age + LVEF	101 (24)	0.78	0.024	0.75	0.99
NYHA improvement ≥1 class	NYHA class + age + LVEF	101 (23)	0.78	0.034	0.75	0.97
NYHA improvement ≥1 class	TAPSE + age + LVEF	99 (22)	0.81	0.031	0.77	0.94
NYHA improvement ≥1 class	sPAP + age + LVEF	83 (19)	0.74	0.043	0.70	0.89
Broader composite endpoint (Cox)	Age + LVEF + NYHA class	101 (47)	0.59	0.039	0.56	0.79

Endpoint	Model	n (events)	Apparent C	Optimism	Optimism-corrected C	Corrected calibration slope
Death or HF hospitalisation (Cox)	Age + LVEF + NYHA class	101 (30)	0.54	0.082	0.46	0.23

Table S6. Sensitivity analyses restricted to patients with HFrEF (n = 94) and after exclusion of patients with CRT-D (n = 82).

(A) Paired comparisons

Parameter	n	Baseline	6 months	p-value
**All patients (N = 101)**				
LVEF	101	28.8 (24.4–36.0)	31.6 (25.6–38.0)	<0.001
LVEDV	101	197 (135–261)	182 (125–249)	<0.001
LVESV	101	138 (91–197)	124 (76–185)	<0.001
LV GLS	99	-8.9 (-11.9–-6.7)	-9.9 (-12.4–-7.2)	0.003
sPAP	69	33 (24–40)	31 (23–35)	<0.001
TAPSE/sPAP	68	0.78 (0.54–0.85)	0.79 (0.61–0.90)	<0.001
**HFrEF only (N = 94)**				
LVEF	94	27.5 (24.2–34.2)	31.4 (25.7–38.0)	<0.001
LVEDV	94	210 (141–269)	182 (128–255)	<0.001
LVESV	94	148 (95–203)	125 (77–190)	<0.001
LV GLS	92	-8.9 (-11.7–-6.6)	-10.0 (-12.4–-7.1)	<0.001
sPAP	66	33 (24–41)	31 (23–35)	<0.001
TAPSE/sPAP	65	0.77 (0.54–0.84)	0.79 (0.62–0.90)	<0.001
**Without CRT-D (N = 82)**				
LVEF	82	29.6 (24.9–36.9)	31.6 (27.0–39.0)	0.002
LVEDV	82	189 (134–248)	174 (124–227)	<0.001
LVESV	82	129 (88–184)	120 (75–164)	<0.001
LV GLS	82	-9.1 (-11.9–-7.1)	-10.0 (-12.4–-7.5)	0.057
sPAP	56	31 (24–42)	30 (23–36)	<0.001
TAPSE/sPAP	55	0.80 (0.54–0.85)	0.82 (0.58–0.90)	<0.001

(B) Response endpoints, n (%)

Population	N	Operational reverse remodelling	LVESV reduction ≥15%	LVEF increase ≥5 points	NYHA improvement
All patients	101	74 (73.3%)	56 (55.4%)	24 (23.8%)	23 (22.8%)
HFrEF only	94	73 (77.7%)	55 (58.5%)	24 (25.5%)	22 (23.4%)
Without CRT-D	82	58 (70.7%)	45 (54.9%)	18 (22.0%)	18 (22.0%)

(C) Firth logistic regression adjusted for age and baseline LVEF

Endpoint	Variable	Increment	n (events)	Adjusted OR (95% CI)	p-value
**HFrEF only**					
Operational LV reverse remodelling	BMI	per 1 kg/m²	94 (73)	1.31 (1.08–1.57)	0.005
Operational LV reverse remodelling	Anaemia	yes vs no	94 (73)	2.00 (0.61–6.54)	0.252
Operational LV reverse remodelling	LVEDV	per 10 mL	94 (73)	1.38 (1.19–1.61)	<0.001
Operational LV reverse remodelling	LV GLS magnitude (|GLS|)	per 1% (higher = better)	92 (71)	0.92 (0.73–1.16)	0.460
LVESV reduction ≥15%	LVEDV	per 10 mL	94 (55)	1.12 (1.03–1.22)	0.009
LVESV reduction ≥15%	LV GLS magnitude (|GLS|)	per 1% (higher = better)	92 (54)	1.18 (0.96–1.46)	0.123

Endpoint	Variable	Increment	n (events)	Adjusted OR (95% CI)	p-value
LVEF increase ≥5 points	TAPSE	per 1 mm	92 (22)	0.66 (0.53–0.81)	<0.001
LVEF increase ≥5 points	sPAP	per 5 mmHg	80 (16)	1.29 (1.00–1.67)	0.050
NYHA improvement ≥1 class	NYHA class	per 1 class	94 (22)	5.29 (1.84–15.16)	0.002
NYHA improvement ≥1 class	TAPSE	per 1 mm	92 (21)	0.79 (0.67–0.93)	0.005
NYHA improvement ≥1 class	sPAP	per 5 mmHg	80 (19)	1.37 (1.04–1.82)	0.027
**Without CRT-D**					
Operational LV reverse remodelling	BMI	per 1 kg/m²	82 (58)	1.35 (1.11–1.64)	0.002
Operational LV reverse remodelling	Anaemia	yes vs no	82 (58)	2.71 (0.71–10.27)	0.144
Operational LV reverse remodelling	LVEDV	per 10 mL	82 (58)	1.35 (1.15–1.58)	<0.001
Operational LV reverse remodelling	LV GLS magnitude (|GLS|)	per 1% (higher = better)	82 (58)	0.97 (0.77–1.21)	0.758
LVESV reduction ≥15%	LVEDV	per 10 mL	82 (45)	1.11 (1.01–1.21)	0.029
LVESV reduction ≥15%	LV GLS magnitude (|GLS|)	per 1% (higher = better)	82 (45)	1.19 (0.96–1.47)	0.112
LVEF increase ≥5 points	TAPSE	per 1 mm	80 (16)	0.68 (0.55–0.85)	<0.001
LVEF increase ≥5 points	sPAP	per 5 mmHg	66 (12)	1.36 (0.99–1.87)	0.059
NYHA improvement ≥1 class	NYHA class	per 1 class	82 (18)	6.64 (2.07–21.25)	0.001
NYHA improvement ≥1 class	TAPSE	per 1 mm	80 (17)	0.85 (0.71–1.01)	0.063
NYHA improvement ≥1 class	sPAP	per 5 mmHg	66 (15)	1.73 (1.15–2.58)	0.008

Table S7. Baseline characteristics associated with LVESV reduction ≥15%: Firth penalised logistic regression († adjusted for age and baseline LVEF; ‡ adjusted for the other core covariate only).

Variable	Increment	Unadjusted OR (95% CI)	p	Adjusted OR (95% CI) †	p	n (events)
Age	per 10 years	1.01 (0.76–1.34)	0.927	1.05 (0.78–1.40)‡	0.756	101 (56)
Male sex	yes vs no	1.32 (0.51–3.40)	0.567	1.30 (0.50–3.38)	0.589	101 (56)
BMI	per 1 kg/m²	1.34 (1.15–1.55)	<0.001	1.34 (1.15–1.55)	<0.001	101 (56)
Arterial hypertension	yes vs no	1.08 (0.49–2.38)	0.841	1.13 (0.49–2.59)	0.778	101 (56)
Diabetes mellitus	yes vs no	1.16 (0.40–3.33)	0.784	1.16 (0.39–3.47)	0.784	101 (56)
Atrial fibrillation	yes vs no	1.62 (0.69–3.81)	0.266	1.66 (0.70–3.93)	0.250	101 (56)
Anaemia	yes vs no	1.76 (0.72–4.29)	0.215	1.62 (0.66–4.01)	0.296	101 (56)
Ischaemic aetiology	yes vs no	1.28 (0.57–2.91)	0.551	1.32 (0.56–3.11)	0.519	101 (56)
NYHA class	per 1 class	0.73 (0.39–1.36)	0.314	0.56 (0.28–1.14)	0.113	101 (56)
Systolic BP	per 10 mmHg	0.84 (0.66–1.08)	0.169	0.84 (0.65–1.09)	0.186	101 (56)
Heart rate	per 10 bpm	0.79 (0.55–1.12)	0.189	0.78 (0.54–1.12)	0.173	99 (54)
Haemoglobin	per 1 g/dL	0.90 (0.70–1.15)	0.405	0.91 (0.71–1.17)	0.450	100 (56)
eGFR	per 10 mL/min/1.73 m²	0.97 (0.81–1.16)	0.756	0.97 (0.79–1.19)	0.756	100 (56)
LVEF	per 5%	0.85 (0.64–1.13)	0.258	0.85 (0.64–1.12)‡	0.247	101 (56)
LVEDV	per 10 mL	1.06 (1.01–1.12)	0.021	1.09 (1.01–1.18)	0.028	101 (56)
LAV	per 10 mL	1.08 (0.98–1.19)	0.129	1.07 (0.96–1.18)	0.206	96 (54)
E/e′	per 1 unit	0.98 (0.89–1.07)	0.603	0.95 (0.85–1.05)	0.326	95 (52)
Diastolic dysfunction grade	per 1 grade	1.56 (0.77–3.14)	0.219	1.48 (0.72–3.08)	0.289	88 (50)
TAPSE	per 1 mm	0.84 (0.75–0.94)	0.002	0.81 (0.71–0.92)	0.001	99 (55)
sPAP	per 5 mmHg	0.82 (0.66–1.02)	0.079	0.83 (0.66–1.03)	0.095	83 (48)
LV GLS magnitude (|GLS|)	per 1% (higher = better)	1.03 (0.91–1.16)	0.612	1.20 (0.98–1.47)	0.072	99 (55)
Mitral regurgitation grade	per 1 grade	1.37 (0.83–2.27)	0.217	1.33 (0.79–2.23)	0.286	101 (56)
CRT-D	yes vs no	1.12 (0.41–3.06)	0.832	0.90 (0.30–2.66)	0.843	101 (56)
ICD	yes vs no	0.55 (0.25–1.23)	0.145	0.52 (0.23–1.17)	0.116	101 (56)

Table S8. Baseline characteristics associated with LVESV reduction ≥10%: Firth penalised logistic regression († adjusted for age and baseline LVEF; ‡ adjusted for the other core covariate only).

Variable	Increment	Unadjusted OR (95% CI)	p	Adjusted OR (95% CI) †	p	n (events)
Age	per 10 years	0.89 (0.65–1.21)	0.452	0.97 (0.70–1.33)‡	0.833	101 (69)
Male sex	yes vs no	1.33 (0.49–3.57)	0.574	1.36 (0.49–3.80)	0.560	101 (69)
BMI	per 1 kg/m²	1.31 (1.12–1.53)	<0.001	1.34 (1.13–1.58)	<0.001	101 (69)
Arterial hypertension	yes vs no	0.90 (0.39–2.10)	0.815	1.12 (0.45–2.80)	0.815	101 (69)
Diabetes mellitus	yes vs no	1.09 (0.35–3.36)	0.885	1.27 (0.37–4.33)	0.702	101 (69)
Atrial fibrillation	yes vs no	1.33 (0.54–3.31)	0.537	1.52 (0.57–4.01)	0.400	101 (69)
Anaemia	yes vs no	1.61 (0.61–4.25)	0.336	1.32 (0.48–3.62)	0.587	101 (69)
Ischaemic aetiology	yes vs no	0.94 (0.40–2.23)	0.888	1.10 (0.43–2.80)	0.839	101 (69)
NYHA class	per 1 class	0.87 (0.45–1.69)	0.688	0.51 (0.23–1.13)	0.097	101 (69)
Systolic BP	per 10 mmHg	0.87 (0.67–1.13)	0.305	0.93 (0.70–1.23)	0.602	101 (69)
Heart rate	per 10 bpm	0.81 (0.57–1.15)	0.234	0.77 (0.52–1.14)	0.191	99 (67)
Haemoglobin	per 1 g/dL	0.90 (0.69–1.17)	0.414	0.90 (0.68–1.19)	0.469	100 (69)
eGFR	per 10 mL/min/1.73 m²	0.95 (0.78–1.15)	0.602	0.87 (0.69–1.11)	0.263	100 (69)
LVEF	per 5%	0.64 (0.47–0.87)	0.005	0.64 (0.47–0.89)‡	0.007	101 (69)
LVEDV	per 10 mL	1.15 (1.07–1.23)	<0.001	1.16 (1.06–1.27)	0.002	101 (69)
LAV	per 10 mL	1.15 (1.03–1.29)	0.015	1.13 (1.00–1.26)	0.046	96 (64)
E/e′	per 1 unit	0.98 (0.89–1.08)	0.683	0.93 (0.83–1.04)	0.196	95 (65)
Diastolic dysfunction grade	per 1 grade	1.45 (0.66–3.16)	0.350	1.14 (0.51–2.52)	0.750	88 (63)
TAPSE	per 1 mm	0.90 (0.80–1.00)	0.056	0.84 (0.74–0.97)	0.016	99 (68)
sPAP	per 5 mmHg	0.81 (0.64–1.02)	0.074	0.78 (0.61–0.99)	0.039	83 (61)
LV GLS magnitude (∣GLS∣)	per 1% (higher = better)	0.89 (0.78–1.02)	0.084	1.03 (0.84–1.26)	0.751	99 (68)
Mitral regurgitation grade	per 1 grade	1.20 (0.70–2.03)	0.507	1.12 (0.64–1.96)	0.701	101 (69)
CRT-D	yes vs no	1.31 (0.43–3.95)	0.636	0.80 (0.23–2.78)	0.729	101 (69)
ICD	yes vs no	0.68 (0.29–1.57)	0.367	0.52 (0.21–1.31)	0.166	101 (69)

Table S9. ANCOVA models of the 6-month value adjusted for the baseline value of the same parameter and for age. For LV volumes (log-transformed), estimates are percentage differences in the 6-month volume per increment; for LVEF, differences in percentage points.

Predictor	Increment	n	Estimate (95% CI)	p-value
**Follow-up LVESV (% difference)**				
BMI	per 1 kg/m²	101	-0.9 (-3.3–1.5)	0.457
Anaemia	yes vs no	101	-15.1 (-28.8–1.3)	0.068
LV GLS magnitude (∣GLS∣)	per 1% (higher = better)	99	-5.6 (-8.8–-2.3)	0.001
TAPSE	per 1 mm	99	6.4 (4.5–8.3)	<0.001
sPAP	per 5 mmHg	83	4.0 (0.4–7.8)	0.030
NYHA class	per 1 class	101	25.3 (10.4–42.2)	<0.001
**Follow-up LVEDV (% difference)**				
BMI	per 1 kg/m²	101	-1.1 (-3.0–0.9)	0.277
Anaemia	yes vs no	101	-8.7 (-20.8–5.3)	0.209
LV GLS magnitude (∣GLS∣)	per 1% (higher = better)	99	-4.7 (-7.1–-2.3)	<0.001
TAPSE	per 1 mm	99	4.5 (2.9–6.1)	<0.001
sPAP	per 5 mmHg	83	3.7 (0.7–6.8)	0.015
NYHA class	per 1 class	101	22.9 (11.5–35.5)	<0.001
**Follow-up LVEF (percentage points)**				
BMI	per 1 kg/m²	101	-0.2 (-0.7–0.3)	0.413
Anaemia	yes vs no	101	3.8 (0.3–7.3)	0.033
LV GLS magnitude (∣GLS∣)	per 1% (higher = better)	99	1.3 (0.6–1.9)	<0.001

Predictor	Increment	n	Estimate (95% CI)	p-value
TAPSE	per 1 mm	99	-1.2 (-1.5–-0.9)	<0.001
sPAP	per 5 mmHg	83	-0.4 (-1.2–0.3)	0.262
NYHA class	per 1 class	101	-3.1 (-5.8–-0.4)	0.023
LVEDV	per 10 mL	101	-0.6 (-0.8–-0.3)	<0.001

Table S10. Unadjusted Firth logistic regression for combined echocardiographic and functional response (ΔLVEF ≥5 points and NYHA improvement; n = 8). Given the very small number of events, no adjusted model was fitted.

Variable	Unit	n (events)	Unadjusted Firth OR (95% CI)	p-value
NYHA class	per 1 class	101 (8)	12.87 (2.18–76.01)	0.005
E/e′	per 1 unit	95 (8)	1.15 (1.01–1.32)	0.037
Diastolic dysfunction grade	per 1 grade	88 (7)	3.01 (0.97–9.39)	0.057
TAPSE	per 1 mm	99 (7)	0.71 (0.56–0.90)	0.005
sPAP	per 5 mmHg	83 (5)	1.46 (0.99–2.14)	0.054
Mitral regurgitation grade	per 1 grade	101 (8)	3.01 (1.22–7.44)	0.017
LVEF	per 5%	101 (8)	0.63 (0.34–1.19)	0.156
LV GLS magnitude (|GLS|)	per 1% (higher = better)	99 (8)	0.96 (0.78–1.19)	0.732

Table S11. Baseline characteristics and death or heart failure hospitalisation after the 6-month visit (Cox regression; 30 events). Multivariable model: age, baseline LVEF and NYHA class.

Variable	Increment	n (events)	Univariable HR (95% CI)	p	Multivariable HR (95% CI)	p
Age	per 10 years	101 (30)	1.06 (0.81–1.39)	0.649	1.03 (0.78–1.37)	0.811
Male sex	yes vs no	101 (30)	1.53 (0.59–4.01)	0.384		
BMI	per 1 kg/m²	101 (30)	1.06 (0.95–1.18)	0.320		
Arterial hypertension	yes vs no	101 (30)	1.86 (0.87–3.97)	0.110		
Diabetes mellitus	yes vs no	101 (30)	0.63 (0.22–1.80)	0.385		
Atrial fibrillation	yes vs no	101 (30)	1.01 (0.48–2.13)	0.980		
Anaemia	yes vs no	101 (30)	0.53 (0.22–1.30)	0.168		
Ischaemic aetiology	yes vs no	101 (30)	2.39 (1.16–4.92)	0.018		
NYHA class	per 1 class	101 (30)	0.99 (0.55–1.77)	0.968	1.08 (0.56–2.08)	0.812
Systolic BP	per 10 mmHg	101 (30)	1.10 (0.89–1.36)	0.386		
Heart rate	per 10 bpm	99 (29)	1.17 (0.86–1.60)	0.317		
Haemoglobin	per 1 g/dL	100 (29)	1.08 (0.88–1.33)	0.468		
eGFR	per 10 mL/min/1.73 m²	100 (29)	0.97 (0.82–1.15)	0.753		
LVEF	per 5%	101 (30)	1.14 (0.88–1.48)	0.333	1.15 (0.86–1.53)	0.352
LVEDV	per 10 mL	101 (30)	1.00 (0.95–1.04)	0.866		
LAV	per 10 mL	96 (30)	1.03 (0.95–1.11)	0.539		
E/e′	per 1 unit	95 (29)	1.00 (0.92–1.09)	0.995		
Diastolic dysfunction grade	per 1 grade	88 (28)	0.52 (0.26–1.05)	0.068		
TAPSE	per 1 mm	99 (30)	0.94 (0.86–1.04)	0.251		
sPAP	per 5 mmHg	83 (28)	1.07 (0.89–1.28)	0.479		
LV GLS magnitude (|GLS|)	per 1% (higher = better)	99 (29)	0.98 (0.88–1.10)	0.783		
Mitral regurgitation grade	per 1 grade	101 (30)	1.00 (0.65–1.53)	0.998		
CRT-D	yes vs no	101 (30)	0.53 (0.16–1.76)	0.298		

Variable	Increment	n (events)	Univariable HR (95% CI)	p	Multivariable HR (95% CI)	p
ICD	yes vs no	101 (30)	0.82 (0.40–1.70)	0.595		

Table S12. Broader composite endpoint (all-cause death, heart failure hospitalisation, non-fatal MI/PCI, incident AF and ventricular arrhythmias) after the 6-month visit. † Ventricular arrhythmias were assessed only in patients with available device telemetry or Holter monitoring (n = 55); § incident AF was assessed in patients without a history of AF (n = 68). Multivariable model: age, baseline LVEF and NYHA class (n = 101, 47 events; proportional hazards global test p = 0.90).

(A) Broader composite endpoint and its components

Event	n (%)
Follow-up after the 6-month visit, months, median (IQR)	32 (25–33)
Broader composite endpoint	47 (46.5%)
All-cause death	14 (13.9%)
HF hospitalisation	18 (17.8%)
Non-fatal MI/PCI	2 (2.0%)
Incident atrial fibrillation §	14/68 (20.6%)
Ventricular arrhythmias (NSVT/VT/VF) †	14/55 (25.5%)

(B) Cox regression for the broader composite endpoint

Variable	Increment	n (events)	Univariable HR (95% CI)	p	Multivariable HR (95% CI)	p
Age	per 10 years	101 (47)	1.17 (0.94–1.45)	0.166	1.11 (0.88–1.39)	0.382
Male sex	yes vs no	101 (47)	1.32 (0.64–2.72)	0.460		
BMI	per 1 kg/m²	101 (47)	0.99 (0.91–1.09)	0.891		
Arterial hypertension	yes vs no	101 (47)	1.82 (0.99–3.34)	0.052		
Diabetes mellitus	yes vs no	101 (47)	0.73 (0.32–1.63)	0.438		
Atrial fibrillation	yes vs no	101 (47)	0.71 (0.38–1.34)	0.295		
Anaemia	yes vs no	101 (47)	0.91 (0.49–1.71)	0.775		
Ischaemic aetiology	yes vs no	101 (47)	1.42 (0.79–2.56)	0.237		
NYHA class	per 1 class	101 (47)	0.99 (0.62–1.59)	0.964	1.16 (0.68–1.97)	0.585
Systolic BP	per 10 mmHg	101 (47)	1.04 (0.88–1.24)	0.614		
Heart rate	per 10 bpm	99 (46)	1.00 (0.75–1.33)	0.982		
Haemoglobin	per 1 g/dL	100 (46)	1.00 (0.85–1.18)	0.964		
eGFR	per 10 mL/min/1.73 m²	100 (46)	0.99 (0.86–1.13)	0.832		
LVEF	per 5%	101 (47)	1.31 (1.06–1.60)	0.011	1.31 (1.05–1.64)	0.016
LVEDV	per 10 mL	101 (47)	0.96 (0.92–1.00)	0.033		
LAV	per 10 mL	96 (47)	0.98 (0.92–1.05)	0.638		
E/e′	per 1 unit	95 (46)	1.01 (0.94–1.08)	0.811		
Diastolic dysfunction grade	per 1 grade	88 (42)	0.55 (0.31–0.95)	0.033		
TAPSE	per 1 mm	99 (46)	0.95 (0.88–1.03)	0.234		
sPAP	per 5 mmHg	83 (42)	0.97 (0.83–1.14)	0.719		
LV GLS magnitude (|GLS|)	per 1% (higher = better)	99 (46)	1.04 (0.96–1.14)	0.313		
Mitral regurgitation grade	per 1 grade	101 (47)	1.01 (0.72–1.41)	0.974		
CRT-D	yes vs no	101 (47)	0.84 (0.37–1.88)	0.666		

Variable	Increment	n (events)	Univariable HR (95% CI)	p	Multivariable HR (95% CI)	p
ICD	yes vs no	101 (47)	0.77 (0.43–1.37)	0.367		

Table S13. Multivariable Cox model (age, baseline LVEF and NYHA class) for death or heart failure hospitalisation, the broader composite endpoint and the broader composite endpoint excluding ventricular arrhythmias (time origin: 6-month visit).

Variable	Increment	HR (95% CI)	p-value
**Death or HF hospitalisation (30 events)**			
Age	per 10 years	1.03 (0.78–1.37)	0.811
NYHA class	per 1 class	1.08 (0.56–2.08)	0.812
LVEF	per 5%	1.15 (0.86–1.53)	0.352
**Broader composite endpoint (47 events)**			
Age	per 10 years	1.11 (0.88–1.39)	0.382
NYHA class	per 1 class	1.16 (0.68–1.97)	0.585
LVEF	per 5%	1.31 (1.05–1.64)	0.016
**Broader composite excluding ventricular arrhythmias (42 events)**			
Age	per 10 years	1.10 (0.86–1.40)	0.449
NYHA class	per 1 class	1.04 (0.60–1.82)	0.887
LVEF	per 5%	1.26 (0.99–1.60)	0.057

Table S14. Association between baseline LVEF and the individual components of the broader composite endpoint (univariable Cox regression; time origin: 6-month visit). Incident atrial fibrillation was analysed in patients without a history of atrial fibrillation (n = 68); ventricular arrhythmias were assessed only in patients with available device telemetry or Holter monitoring (n = 55).

Component	n (events)	HR per 5% higher LVEF (95% CI)	p-value
All-cause death	101 (14)	1.18 (0.81–1.71)	0.382
HF hospitalisation	101 (18)	1.35 (0.98–1.87)	0.069
Death or HF hospitalisation	101 (30)	1.14 (0.88–1.48)	0.333
Incident atrial fibrillation (patients without history of AF)	68 (14)	1.57 (1.03–2.39)	0.036
Ventricular arrhythmias (monitored patients)	55 (14)	1.17 (0.80–1.73)	0.417

Table S15. Association between 6-month response and the subsequent broader composite endpoint (time origin: 6-month visit). * Adjusted for age and baseline LVEF.

Endpoint	Exposure at 6 months	Events: exposed vs unexposed	Unadjusted HR (95% CI)	Adjusted HR (95% CI) *	p (adj.)	Log-rank p
Broader composite endpoint	LVEF increase ≥5 points	11/24 vs 36/77	1.17 (0.59–2.31)	1.38 (0.69–2.78)	0.364	0.639
Broader composite endpoint	NYHA improvement ≥1 class	13/23 vs 34/78	1.40 (0.74–2.65)	1.73 (0.89–3.37)	0.107	0.307
Broader composite endpoint	Operational LV reverse remodelling	33/74 vs 14/27	0.64 (0.34–1.22)	0.96 (0.46–2.00)	0.915	0.164
Broader composite endpoint	LVESV reduction ≥15%	24/56 vs 23/45	0.75 (0.42–1.36)	0.82 (0.45–1.49)	0.513	0.352

Table S16. Association between 6-month response and the broader composite endpoint excluding ventricular arrhythmias (time origin: 6-month visit). * Adjusted for age and baseline LVEF.

Endpoint	Exposure at 6 months	Events: exposed vs unexposed	Unadjusted HR (95% CI)	Adjusted HR (95% CI) *	p (adj.)	Log-rank p
Broader composite excluding ventricular arrhythmias	LVEF increase ≥5 points	9/24 vs 33/77	1.05 (0.50–2.21)	1.21 (0.57–2.60)	0.619	0.867
Broader composite excluding ventricular arrhythmias	NYHA improvement ≥1 class	11/23 vs 31/78	1.30 (0.65–2.58)	1.56 (0.76–3.18)	0.222	0.455
Broader composite excluding ventricular arrhythmias	Operational LV reverse remodelling	30/74 vs 12/27	0.67 (0.34–1.32)	0.95 (0.43–2.09)	0.906	0.240
Broader composite excluding ventricular arrhythmias	LVESV reduction ≥15%	21/56 vs 21/45	0.69 (0.37–1.29)	0.74 (0.39–1.40)	0.358	0.263

Table S17. Paired changes in LV volumes and LVEF in the 26 patients with complete therapy data and no documented initiation or discontinuation of β-blockers, MRAs, ACE inhibitors, ARBs or ARNI between baseline and 6 months. Baseline and 6-month values are median (IQR) at each time point; changes are medians (IQR) of individual paired changes; Wilcoxon signed-rank test. This analysis was specified for the revised manuscript.

Parameter	n	Baseline	6 months	Median paired change (IQR)	p-value
LVEDV (mL)	26	227.0 (121.1–315.0)	185.0 (131.0–266.0)	−12.6% (−20.7 to 5.6)	0.029
LVESV (mL)	26	167.6 (82.5–245.9)	132.6 (88.2–199.6)	−16.1% (−26.1 to −4.8)	0.022
LVEF (%)	26	26.9 (22.9–37.2)	28.9 (24.7–35.2)	2.0 points (−1.3 to 3.0)	0.253
Operational LV reverse remodelling, n (%)	26		19 (73.1%)		
LVESV reduction ≥15%, n (%)	26		14 (53.8%)		
LVEF increase ≥5 points, n (%)	26		5 (19.2%)		
